# ClpXP protease targets long-lived DNA translocation states of a helicase-like motor to cause restriction alleviation

**DOI:** 10.1093/nar/gku851

**Published:** 2014-09-26

**Authors:** Michelle Simons, Fiona M. Diffin, Mark D. Szczelkun

**Affiliations:** DNA-Protein Interactions Unit, School of Biochemistry, University of Bristol, Bristol, BS8 1TD, UK

## Abstract

We investigated how *Escherichia coli* ClpXP targets the helicase-nuclease (HsdR) subunit of the bacterial Type I restriction–modification enzyme EcoKI during restriction alleviation (RA). RA is a temporary reduction in endonuclease activity that occurs when Type I enzymes bind unmodified recognition sites on the host genome. These conditions arise upon acquisition of a new system by a naïve host, upon generation of new sites by genome rearrangement/mutation or during homologous recombination between hemimethylated DNA. Using recombinant DNA and proteins *in vitro*, we demonstrate that ClpXP targets EcoKI HsdR during dsDNA translocation on circular DNA but not on linear DNA. Protein roadblocks did not activate HsdR proteolysis. We suggest that DNA translocation lifetime, which is elevated on circular DNA relative to linear DNA, is important to RA. To identify the ClpX degradation tag (degron) in HsdR, we used bioinformatics and biochemical assays to design N- and C-terminal mutations that were analysed *in vitro* and *in vivo*. None of the mutants produced a phenotype consistent with loss of the degron, suggesting an as-yet-unidentified recognition pathway. We note that an EcoKI nuclease mutant still produces cell death in a *clpx^−^* strain, consistent with DNA damage induced by unregulated motor activity.

Restriction–modification (RM) systems confer selective advantage to bacterial and archaeal cells, principally by protecting their hosts from infection by foreign DNA elements such as bacteriophages or plasmids. For the RM systems classified as Types I, II and III, the distinction between foreign and self occurs at the level of DNA recognition (1–4); in most cases, the bacterial DNA genome is modified at specific sites by a methyltransferase (MTase) activity whilst incoming DNA, on which the sites are unmodified, is recognized and cleaved by a cognate nuclease activity. Driven by their utility for host survival and against a backdrop of counteracting evolutionary pressures, RM systems are subject to high levels of adaptation and horizontal gene transfer (5,6). However, the acquisition of an RM system with a new DNA specificity presents a problem; how can it establish when the virgin host initially has an unmodified genome which should be recognized as ‘foreign’ and thus subjected to double-stranded DNA (dsDNA) breaks? To prevent these toxic events, RM systems have evolved various control mechanisms that can transiently limit the production of, or activity of, the nuclease until the MTase can establish steady-state modification of the genome. Here we investigated the molecular basis for one such control mechanism; the ClpXP-dependent restriction alleviation (RA) of the *Escherichia coli* K Type I RM enzyme EcoKI.

Type I RM systems are assembled from three protein subunits (2,7): HsdS (which for EcoKI recognizes the specific DNA sequence, 5′-AACnnnnnnGTGC-3′), HsdM (which has N6-adenine MTase activity) and HsdR (which has both nuclease and adenosine triphosphatase (ATPase)/helicase-like motor activities) (Figure 1). Family subgroups (Types 1A, 1B, 1C, etc.) can be defined on the basis of amino acid conservation and subunit complementation, with EcoKI being a representative member of family Type IA (8). All families assemble with the same stoichiometry, although the relative stabilities can vary (9,10).

**Figure 1.**
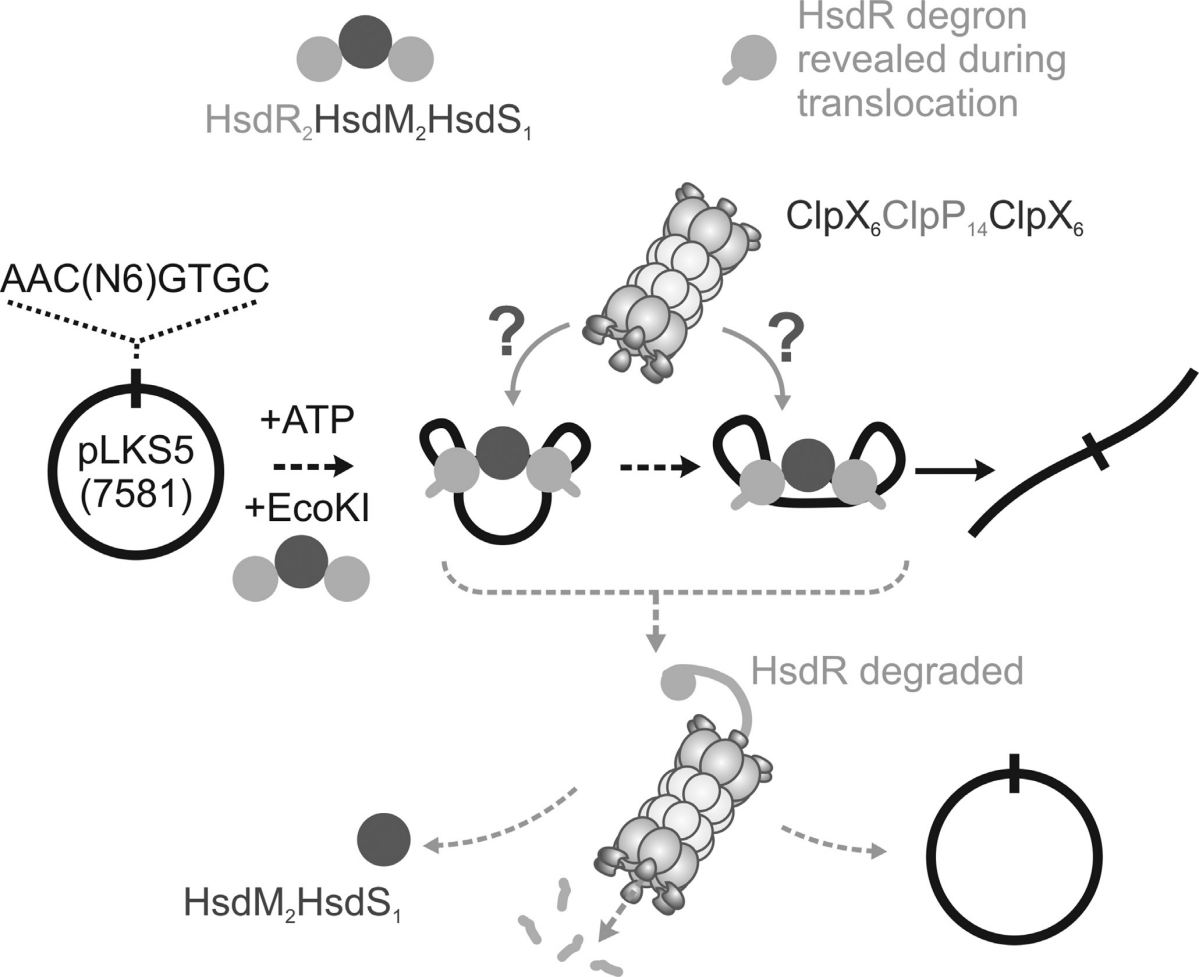
Experimental model for ClpXP-dependent degradation of EcoKI HsdR during translocation and cleavage of circular DNA. The plasmid pLKS5 has a single EcoKI recognition site, as indicated. See the main text for further details.

The most abundant cellular EcoKI complex is an *S*-adenosyl methionine (AdoMet)-dependent DNA MTase with the stoichiometry M_2_S_1_ (9,11). The less abundant ‘holoenzyme’ (R_2_M_2_S_1_) is active as both a DNA MTase and a nuclease, with the balance of activities dependent upon the nature of the DNA. DNA that is hemimethylated (i.e. methylated on one or other strand following replication of the fully modified sites) does not activate the ATPase/helicase-like motor activity but instead elicits maintenance methylation (12). Since EcoKI has an appreciably slower *de novo* methylation activity, fully unmodified sites instead elicit adenosine triphosphate (ATP)-driven bidirectional dsDNA loop translocation that leads to long-range communication between two (or more) enzymes to activate DNA cleavage at distant non-specific sites (13–15). On transferring EcoKI to a naïve *E. coli* host, it takes ∼15 generations before restriction activity can be detected, whereas methylation activity is immediately detected (16). The role of RA is to downregulate the endonuclease activity throughout this interlude (presumably to allow for the slower *de novo* methylation) and thereafter during periods of DNA damage stress when unmodified recognition sites can arise on the host genome (17–28).

RA is induced whenever a Type I holoenzyme encounters an unmodified recognition site ‘on the host genome’; crucially, unmodified sites on foreign DNA (phage genomes, plasmids, etc.) do not induce RA *in vivo*. In contrast to some Type II enzymes (29–31), Type I genes are not under differential transcriptional control (28,32). Instead RA occurs at a post-translational level, principally through a temporary reduction in free HsdR subunits available to assemble into a holoenzyme (although modulation of the MTase activity can also occur) (23,24,26,28,33). The M_2_S_1_ complex that remains can therefore complete the slow maintenance methylation of the unmodified sites. RA is remarkably sensitive, with even small modulations in modification activity impairing the restriction activity (25). RA has been demonstrated for Type IA, IB, IC and ID systems in response to homologous recombination repair of damaged DNA (27), and to acquisition of new systems via transformation, conjugation and transduction (23). In the absence of RA, EcoKI activity on the host DNA rapidly results in dsDNA breaks (34).

RA occurs due to a number of different mechanisms. For EcoKI, RA was demonstrated to be dependent on the subunit-specific proteolysis of HsdR by ClpXP (23). ClpXP is a hetero-oligomeric complex of an AAA+ ATPase (ClpX) and a peptidase (ClpP) (Figure 1) (35). ClpX is a chaperone that recognizes an unstructured peptide sequence (degron) in its substrate (or multiple copies thereof), uses ATP hydrolysis to unfold the tertiary protein structure and feeds the unfolded polypeptide into a proteolytic chamber where ClpP degrades it into small peptide fragments (Figure 1). The ATP-dependent proteases play many important roles in protein homeostasis and control, and are implicated in DNA packaging, repair, replication and transcription (36–41). In an elegant series of genetic and molecular biology assays, Noreen Murray *et al.* demonstrated that ClpXP only targets EcoKI HsdR subunits that are part of a holoenzyme complex capable of ATPase and translocation activity, and that the nuclease activity of the HsdR subunit was not necessary (23–26). ClpXP did not become activated to target HsdR subunits *in trans* (24), arguing against a general SOS response. Rather, each incorrectly located translocation complex is targeted individually.

On the basis of their observations, Murray *et al.* proposed a model in which the initiation of HsdR translocation is accompanied by a conformational change that exposes the degron (Figure 1) (42). However, there is no *a priori* reason to believe that the translocation mechanism on host DNA is any different to that on foreign DNA, yet the latter does not appear to induce RA. To provide further insight into the mechanisms of RA, we examined the effect of ClpXP on EcoKI DNA cleavage activity and HsdR integrity *in vitro* using recombinant, purified components. Our data are consistent with an HsdR–ClpXP interaction that requires long-lived DNA translocation but does not require DNA cleavage. Our observations with certain mutants shed light on how DNA translocation, rather than nucleolytic DNA cleavage, can influence the *in vivo* toxicity of Type I RM enzymes.

Biochemical, bioinformatics and proteomic analysis have identified common amino acids motifs at the extreme N- or C-termini of ClpX substrates (35,36). These signals may be latent, only being exposed upon conformational transitions, e.g. (43). Alternatively an adaptor protein may be required to present the motif to ClpX, e.g. (44). The degron can also be located at an internal site within a protein (45–47), and, in these circumstances, may act more like an adaptor with terminal sequences still playing a role in the initiation of protein unfolding. Such multicomponent recognition may be an important feature of many ClpXP targets. In the Supplementary data, we also describe our attempts to unambiguously identify the degron(s) in EcoKI HsdR. We have ruled out the presence of simple degrons at the HsdR termini, but could not otherwise determine the location of the putative degron(s).

## MATERIALS AND METHODS

### Materials, protein preparations and DNA substrates

Oligodeoxyribonucleotides used in this study are detailed in Supplementary Table S1 (Supplementary data). A plasmid with one EcoKI site and one *res* site for Tn*21* resolvase (pMDS36a-Res2) was generated by removing the 177 bp XhoI-SmaI fragment from pMDS36a (48). A plasmid with the EcoKI removed (pLKS5-K0) was generated by QuikChange site-directed mutagenesis using pLKS5 (49). These DNA, plus pLKS5 (one EcoKI site), pSH1 (one EcoKI site; two *res* sites) (50) and pBR322 (two EcoKI sites) (51), were labelled with [^3^H-methyl] thymidine (52). To prepare linear DNA, pBR322, pMDS36a-Res2 and pSH1 were incubated with AlwNI, HindIII or EcoNI, respectively (New England Biolabs), as instructed by the manufacturer, and the linear DNA purified by phenol/chloroform extraction followed by ethanol precipitation. DNA concentrations were determined from absorbance at 260 nm, assuming an extinction coefficient of 0.02 ml/μg·cm and a DNA molecular weight of 6.6 × 10^5^ Da/kbp.

Wild-type EcoKI MTase and HsdRΔN20 were purified as described previously (53). Recombinant clones of HsdR(D298E) and HsdR(K477R) were produced by QuikChange mutagenesis of pRSF-HsdRΔN20, sequenced and the proteins expressed and purified as for the wild-type HsdR. Holoenzyme was reconstituted immediately before use by mixing MTase and HsdR to produce an R_2_M_2_S_1_ complex, as described previously (53). Tn*21* resolvase was purified as described previously (54). The production of the DNA used in the transformation assays is described in the Supplementary data.

To purify his-tagged ClpX, BL21 (DE3) cells (Novagen) were first freshly transformed with the expression vector pET14b-ClpXHis_6_ (55) and pLysS (Novagen) and subsequently grown in 4 l of Luria Broth at 30°C to an optical density of ∼0.6 at 600 nm. Protein expression was then induced with 0.5-mM IPTG and cells were harvested after 1-h growth at 30°C. The cell paste was resuspended in 40-ml buffer D1 (50-mM Na-Phosphate (pH 8.0), 1-M NaCl, 10% (v/v) glycerol) and cells lysed by sonicating for 10-s bursts for 2 min with intermittent cooling on ice (using a 3-mm-tapered microtip at 30% amplitude and a VCX 750W Vibra-Cell ultrasonic processor, Sonics & Materials, Inc., CT, USA). Cell debris was removed by centrifugation at *r*_av_ = ∼100 000 × *g* for 37 min at 4°C. The supernatant was applied to 1-g Protino Ni-IDA Resin (Macherey-Nagel), washed three times with buffer D1 and batch eluted with three washes of buffer D2 (50-mM Na-Phosphate (pH 8.0), 1-M NaCl, 10% (v/v) glycerol, 1-M Imidazole). Elution fractions were dialyzed into buffer D3 (50-mM Tris (pH 8.0), 10-mM MgCl_2_, 10% (v/v) glycerol, 2-mM DTT) for 2 h and applied to an anion exchange column (MonoQ 5/5GL; GE Healthcare) equilibrated with buffer D4 (50-mM Tris (pH 8.0), 10-mM MgCl_2_, 10% (v/v) glycerol, 2-mM dithiothreitol (DTT), 50-mM KCl). Proteins were eluted with a 20-ml gradient of 50–1000-mM KCl. Fractions containing ClpX (<2 ml) were pooled and applied to a high load 16/60 Superdex 200 column equilibrated with buffer D6 (50-mM Tris (pH 8.0), 10-mM MgCl_2_, 10% (v/v) glycerol, 2-mM DTT, 300-mM NaCl). ClpX containing fractions were pooled and diluted in buffer D3 and applied to a MonoQ 5/5GL column as before. Fractions containing ClpX were pooled and dialyzed into storage buffer (50-mM HEPES-KOH (pH7.5), 25-mM MgCl_2_, 0.1-mM ethylenediaminetetraacetic acid (EDTA) (pH 8.0), 200-mM KCl, 10% (v/v) glycerol). Aliquots were frozen in liquid nitrogen and stored at −80°C.

To purify his-tagged ClpP, Top10 cells (Invitrogen) were freshly transformed with the expression vector pYK133 and pREP-4 (56) and subsequently grown in 4 l of Luria Broth at 37°C to an optical density of ∼0.5 at 600 nm. Protein expression was then induced with 0.5-mM IPTG and cells were harvested after 3-h growth at 30°C. The cell paste was resuspended in 40-ml buffer D1 and cells lysed by sonicating as above for ClpX. Cell debris was removed by centrifugation at *r*_av_ = ∼100 000 × *g* for 37 min at 4°C. The supernatant was applied to 1 g of Protino Ni-IDA Resin, washed three times with buffer D1 and batch eluted with three washes of buffer D2. Elution fractions were dialyzed into buffer D3 for 2 h and then applied to a MonoQ 5/5GL column equilibrated with buffer D4. Bound proteins were eluted with a 20-ml gradient from 50–1000-mM KCl. Fractions containing ClpP were pooled and dialyzed into ClpP storage buffer (50-mM Tris (pH 7.5), 25-mM MgCl2, 0.1-mM EDTA (pH 8.0), 200-mM KCl, 1-mM DTT, 10% (v/v) glycerol) for 2 h. Aliquots were frozen in liquid nitrogen and stored at −80°C.

For both ClpX and ClpP, each sample was considered single-use and was not refrozen upon thawing. Protein concentration was determined using the Bio-Rad Protein Assay according to the manufacturer's instructions, using bovine serum albumin (BSA) as a protein standard (Bio-Rad).

### Reactions with ClpXP

Typical reactions were performed in PD buffer (25-mM HEPES-KOH (pH 7.6), 5-mM MgCl_2_, 5-mM KCl, 15-mM NaCl, 0.0032% (v/v) Triton X-100, 10% (v/v) glycerol) and contained 5-nM DNA, 100-μM *S*-adenosyl methionine, 4-mM ATP, 15-U/ml creatine phosphokinase, 20-mM phosphocreatine, 5-nM EcoKI MTase, 40-nM EcoKI HsdR, 1-μM ClpX and 2-μM ClpP. Reactions were carried out at 30°C and started by addition of ATP. Where indicated, Tn*21* resolvase was diluted before use in 0.1-mM Tris (pH 8.0), 7.5-mM K-Glutamate, 1-μM EDTA (pH 8.0), 0.1-μg/μl BSA and added at 200 nM. At timed intervals following the addition of ATP, aliquots were removed and either mixed 1:1 with sodium dodecyl sulphate (SDS) loading buffer (100-mM Tris-Cl (pH 6.8), 2% (w/v) SDS, 20% (v/v) glycerol, 0.1% (w/v) bromophenol blue) or 2:1 with agarose loading buffer (40% (w/v) sucrose, 0.1-M Tris-Cl (pH 8.0), 1-mM EDTA (pH 8.0), 0.1% (w/v) bromophenol blue). SDS samples were loaded onto 10% (w/v) SDS-polyacrylamide gel electrophoresis (PAGE) gels and run at 200 V for ∼45 min, proteins transferred to polyvinyl difluoride (PVDF) membrane (Millipore) and western blotting performed (see below). Agarose samples were heated at 67°C for 20 min then loaded on 1% (w/v) agarose gels in TAE (40-mM Tris-acetate, 1-mM EDTA, pH 8.3) containing 0.5-μg/ml ethidium bromide and run at 2 V/cm overnight to separate the DNA species. Following electrophoresis DNA bands were excised from the gel and the amount of DNA present quantified by scintillation counting, where appropriate.

### Western blotting

PVDF membranes (Millipore) were pre-wetted in 100% (v/v) methanol for 5 min. The SDS-PAGE gels and PVDF membranes were soaked in transfer buffer (10% (v/v) methanol, 50-mM Tris (pH 8.4)) for 30 min. Proteins were transferred to the PVDF membrane using a BioRad Mini Trans-Blot cell according to manufacturer's instructions. Following transfer, the membrane was blocked overnight in Milk (10% (w/v) Semi-Skimmed Milk Powder (Somerfield)) in phosphate buffered saline. Anti-EcoKI polyclonal antibody was diluted 1:20 000 in 10% (w/v) milk solution and incubated with the membrane for 1.5 h. The membrane was washed three times with phosphate buffered saline supplemented with 0.1% (v/v) Tween 20 for at least 5 min per wash. Goat anti-rabbit horse radish peroxidase-conjugated antibody (Santa Cruz Biotechnology) was diluted 1:5000 in 10% (w/v) milk solution and incubated with the membrane for 1 h. The membrane was washed as before. To generate a chemiluminescent signal, Roche POD Western Blotting substrate was used according to manufacturer's instructions. Visualization was performed by exposing Hyperfilm MP (Amersham) to the blot for 20 s or 1 min (depending on the intensity of the bands) before developing the film with an Agfa Curix 60 film processor. Developed blots were scanned and analysed using the Analysis Toolbox software of ImageQuant (Amersham Biosciences, ImageQuant TL version 2005).

## RESULTS

### The effect of ClpXP on nucleolytic cleavage of circular DNA by EcoKI *in vitro*

We purified EcoKI HsdR and MTase separately from recombinant clones and reconstituted the holoenzyme *in vitro*, as described previously (53). This complex has enzyme activities comparable to holoenzyme assembled *in vivo* (9). Expression from *P*_res_ produces two forms of HsdR: a full-length version (ΔN19) and a version truncated by one amino acid at the N-terminus (ΔN20) (9). For these studies we used a recombinant clone that only produces ΔN20; similar results were observed with ΔN19 (data not shown). For consistency with previous studies, all amino acid numbering is based on the N-terminal methionine of the ΔN19 species. Using recombinant clones kindly provided by Tania Baker (Massachusetts Institute of Technology), the ClpXP protease was reconstituted *in vitro* from his-tagged versions of ClpX and ClpP (55,56), using a modified protocol (the Materials and Methods section).

We were unable to observe any effect of ClpXP on EcoKI using our standard EcoKI reaction buffer (data not shown). We suspected this was due to the sensitivity of ClpXP to salt conditions. Therefore, all subsequent experiments used an established ClpXP buffer, PD (the Materials and Methods section) (57). DNA cleavage by EcoKI was slower in PD than the standard buffer. Sufficient EcoKI holoenzyme was added so that >90% cleavage of a supercoiled DNA was achieved within 1 h. The amount of ClpXP-dependent inhibition of EcoKI cleavage (see below) increased as a function of increasing ClpXP concentration. The concentrations presented were the maximum achievable using available stocks and reaction volumes. We also included AdoMet (necessary for EcoKI nuclease activity) and an enzymatic ATP-recycling system.

**Figure 2.**
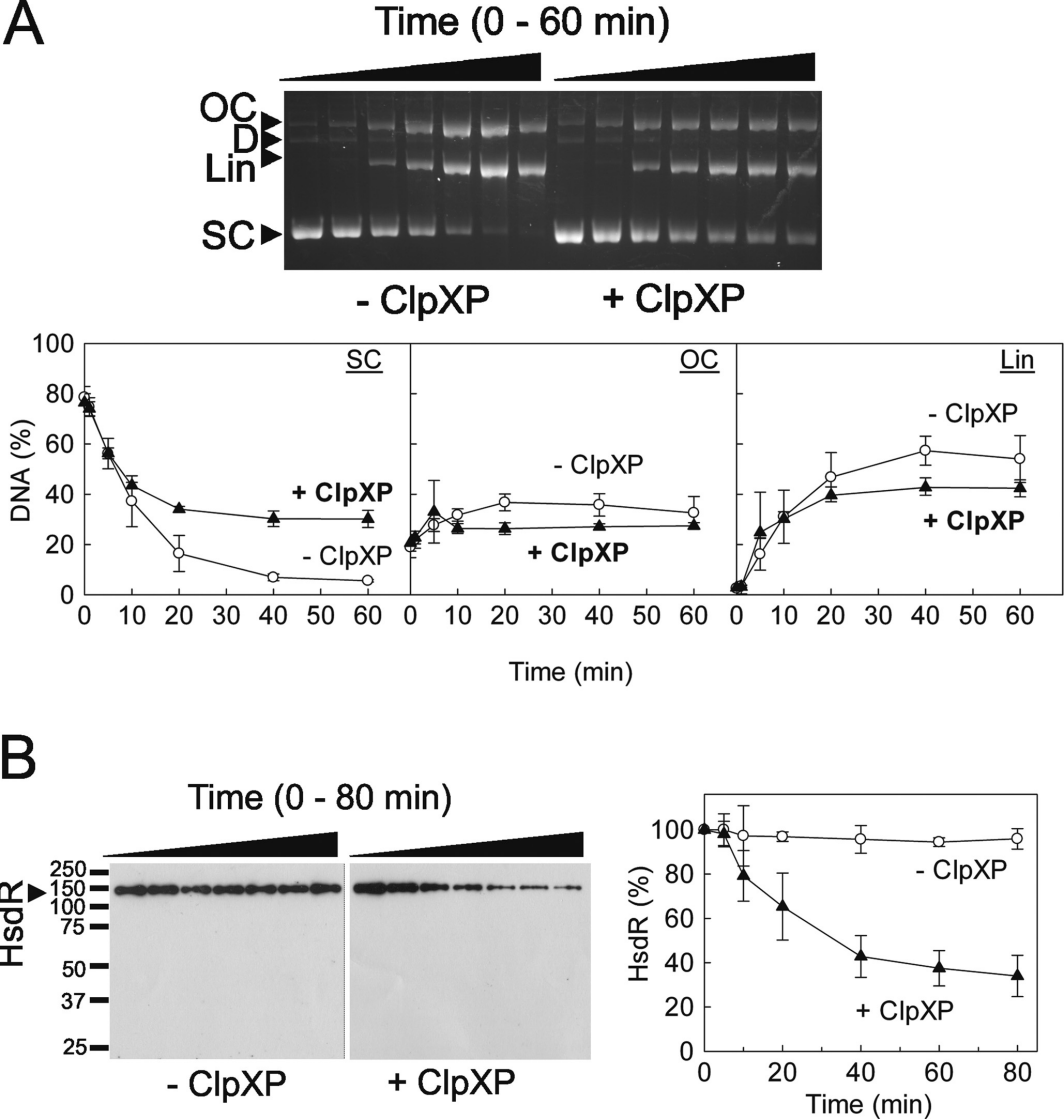
ClpXP degrades HsdR during translocation on circular DNA and inhibits DNA cleavage. (**A**) Cleavage of pLKS5 by EcoKI was monitored as a function of time (0, 5, 10, 20, 40 and 60 min) in the absence or presence of ClpXP. Reactions were initiated by the addition of ATP. Samples were separated by agarose gel electrophoresis: OC, open circle (nicked) plasmid; D, dimeric plasmid; Lin, linear DNA; SC, supercoiled plasmid. Graphs show quantified data: standard deviation error bars from at least three repeat experiments. (**B**) Proteolysis of HsdR was monitored as a function of time (0, 5, 10, 20, 40, 60 and 80 min) in the absence or presence of ClpXP. Samples were separated by SDS gel electrophoresis and blotted using an HsdR antibody. Graphs show quantified data: standard deviation error bars from at least three repeat experiments.

As a DNA substrate, we firstly used a ^3^H-labelled supercoiled plasmid with a single EcoKI site (pLKS1; Figure 1). On one-site circular DNA, the two HsdR motors from a single holoenzyme can translocate around the ring and interact to cleave the DNA (Figure 1A), going via a nicked DNA intermediate to full-length linear DNA. This product is further randomly processed by Type I enzymes to produce smaller linear fragments. The substrates and products were separated by agarose gel electrophoresis and each species quantified by scintillation counting (the processed fragments appear as a smear and were counted as part of the linear band). The endonuclease activity was assessed in the absence and presence of ClpXP (Figure 2A). With the reaction conditions used, ClpXP irreversibly inhibited plasmid cleavage by EcoKI by almost 50%, consistent with its role during RA.

**Figure 3.**
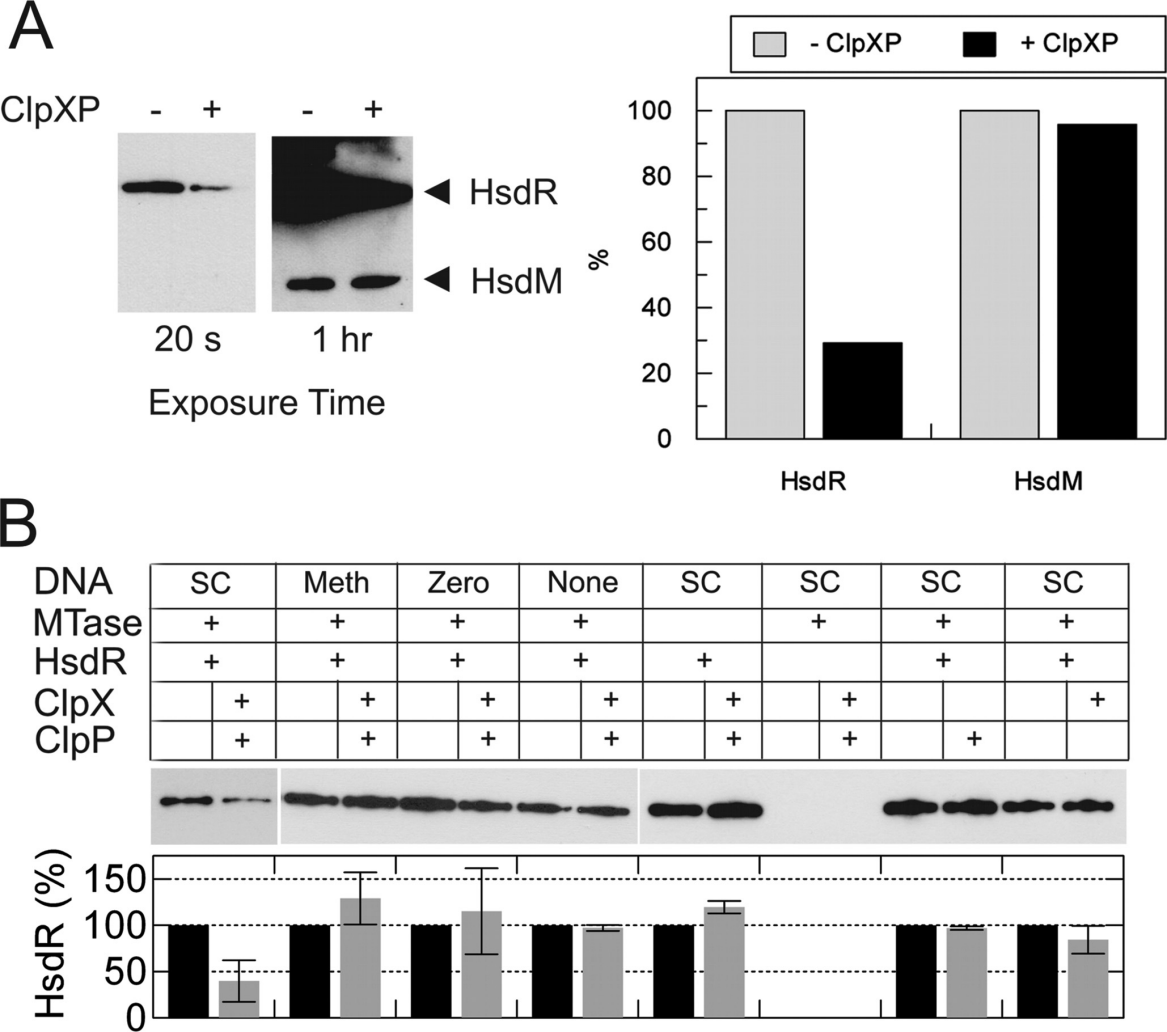
Proteolysis by ClpXP is specific to HsdR and is dependent upon site-specific translocation by the EcoKI holoenzyme. (**A**) Western blot using the polyclonal HsdR antibody for 60-min EcoKI reactions on pLKS5 in the absence or presence of ClpXP. Extended exposure of the blot reveals cross reaction with the HsdM subunit. The graph shows quantitation of the blot. (**B**) Example western blots (break shows different gels) for 60-min reactions under a range of conditions, using, as indicated: supercoiled DNA (SC); DNA pre-methylated at the EcoKI site (Meth); a DNA without an EcoKI site (Zero); or no added DNA (None). Graphs show quantified data: standard deviation error bars from at least three repeat experiments.

To show that the inhibition of EcoKI cleavage by ClpXP was due to proteolysis of the HsdR subunit, the same reaction samples were also analysed by western blotting using an EcoKI polyclonal antibody (kindly provided by Marie Weiserova, Institute of Microbiology, Prague) (58). In the presence of ClpXP, HsdR levels dropped to ∼30% after 1 h (Figure 2B). Intermediate species were not observed, consistent with complete proteolysis by ClpXP following a rate-limiting step. Degradation of the HsdM subunit was not observed (Figure 3A), consistent with the earlier observations *in vivo* that the ClpXP interaction is specific to the HsdR subunit. We note that the incomplete HsdR digestion may be because those HsdR that are irreversibly trapped on the cleaved DNA are in a different conformation that can no longer be recognized by ClpXP. Inhibition of DNA cleavage is likely to occur when the HsdR concentration falls below the threshold for forming the holoenzymes (9). However, the DNA cleavage rate in our *in vitro* assay is faster than the HsdR degradation rate. This suggests that the interaction of ClpXP with HsdR can inhibit the EcoKI cleavage reaction well before actual proteolysis occurs.

### Targeting of EcoKI HsdR by ClpXP is dependent on DNA translocation but does not require DNA cleavage

To further test the model in Figure 1, we used the western blot assay to determine the amount of HsdR degradation after 1 h under different enzyme and substrate conditions (Figure 3B). To test the DNA requirements, we used plasmid DNA substrates with an unmodified EcoKI recognition site (SC), with a site pre-methylated by EcoKI MTase (Meth) and a plasmid DNA without an EcoKI site (Zero). As a control, we also omitted DNA altogether (None). To test the enzyme requirements, we omitted the MTase, the HsdR (as a control for the blotting), ClpX or ClpP. Only with SC DNA, the complete EcoKI holoenzyme and both ClpX and ClpP resulted in significant HsdR degradation.

**Figure 4.**
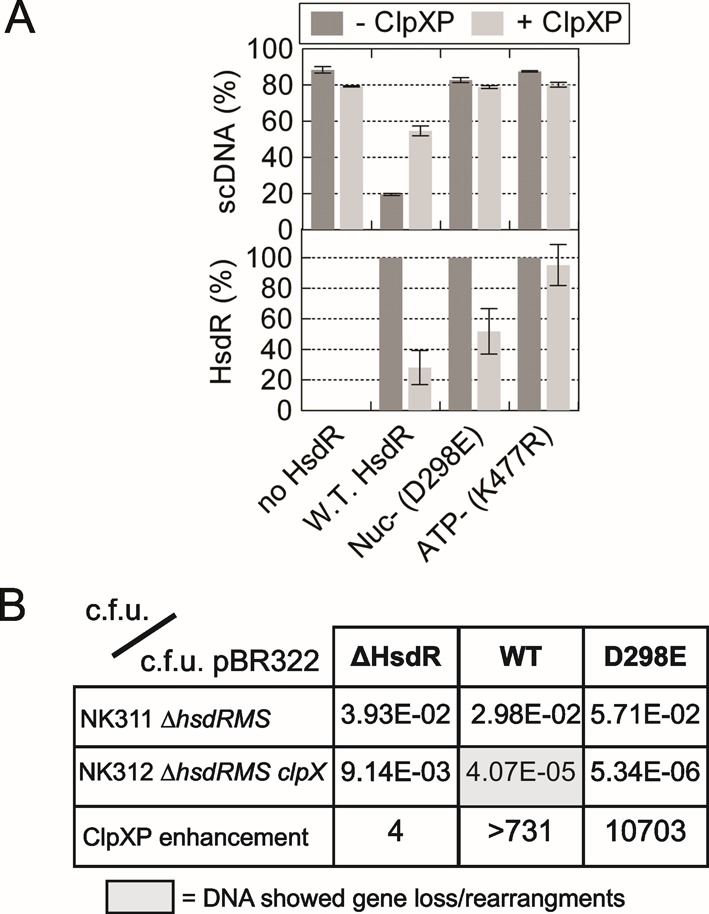
Proteolysis of HsdR is dependent on translocation and not DNA cleavage. (**A**) Quantified data from 60-min reactions on pLKS5 in the presence or absence of ClpXP, and with EcoKI MTase without HsdR, with wild-type HsdR or with HsdRs mutated in either a nuclease (Nuc-) or ATPase (ATP-) motif. Standard deviation error bars from three repeat experiments. (**B**) Transformation screen to measure RA by ClpXP. Colony forming units (c.f.u.) were measured in separate transformation reactions using pBR322 or plasmids carrying the complete EcoKI operon (WT), the genes for the MTase alone (ΔHsdR) or the EcoKI operon with an HsdR mutated within the nuclease domain (D298E). The first two rows show the quotient of the c.f.u. for the EcoKI plasmid and pBR322 in strains with active ClpXP (NK311) or without active ClpXP (NK312). The third row is the quotient of the transformation efficiency in the presence and absence of ClpXP, with elevated values indicating ClpXP-dependent RA. The grey box indicates that plasmid preparations from the successful transformants showed loss of DNA in 100% of cases examined. The smaller DNA could retransform NK312 more efficiently than the original DNA, indicating loss of EcoKI activity.

To explore the consistency between our *in vitro* results and the previous *in vivo* observations, we also examined the effect of two different HsdR mutants on ClpXP activity (Figure 4). HsdR(D298E) is mutated in Motif II of the RecB-family nuclease domain. A holoenzyme formed with this HsdR (Nuc-) cannot cleave DNA but can hydrolyze ATP and translocate similarly to wild-type HsdR (15,59,60). HsdR(K477R) is mutated in Motif I/Walker A box of the Superfamily 2 helicase domain. A holoenzyme formed with this HsdR (ATP-) cannot hydrolyze ATP, and thus neither translocates on nor cleaves DNA (61,62). Neither mutant could cleave DNA above background (Figure 4A, upper panel). HsdR(D298E) was still degraded by ClpXP, whilst HsdR(K477R) was not degraded, within error (Figure 4A, lower panel). These results match the observations *in vivo* with the same mutants (24,26) and are consistent with a requirement for DNA translocation rather than DNA cleavage (42).

We tested RA *in vivo* using a transformation efficiency assay (the method is described in more detail in the Supplementary data). In brief, ClpX+ and ClpX− strains of an EcoKI naïve host were transformed with multi-copy plasmids carrying either the wild-type EcoKI operon, an operon with a truncated/inactivated *hsdr*, or an operon with the D298A mutation within HsdR. The quotient of the number of colony forming units (c.f.u.) with each plasmid and with pBR322 gives a measure of the effect of EcoKI on the transformation efficiency; i.e. where EcoKI activity on the genome leads to DNA damage, there is reduced cell viability and a lower transformation efficiency relative to selection with pBR322. By comparing the ClpX+ and ClpX− strains, we can record the effect of ClpXP as an enhancement value (Figure 4B).

In the presence of EcoKI MTase alone (ΔHsdR), there was a 4-fold difference between the strains. However, in the presence of the holoenzyme (WT), transformation efficiency was markedly reduced in the absence of ClpX, with a >700-fold difference between the strains, consistent with RA in the ClpX+ host. The few surviving ClpX− cells all had DNA rearrangements of the EcoKI plasmid that presumably reflect selective loss of EcoKI activity. Surprisingly, the nuclease mutant of EcoKI (D298A) also produced a marked reduction in transformation efficiency in the absence of ClpX, with an even larger (>10 000-fold) ClpXP enhancement. This is consistent with RA being activated by the motor activity alone as seen previously and above, but also demonstrates that EcoKI is toxic to naïve hosts even in the absence of nuclease activity. Presumably this reflects interference with cellular process by the motor activity. The greater toxicity of the nuclease mutant suggested by the enhanced ClpXP enhancement may reflect changes in the motor caused by mutations in the attached nuclease domain, which can include: decreased translocation and ATPase rates; multiple enzyme populations with different characteristic translocation rates; a tendency to stall during initiation; and altered HsdR turnover dynamics (63).

### Cleavage of linear DNA substrates by EcoKI did not induce HsdR degradation by ClpXP

**Figure 5.**
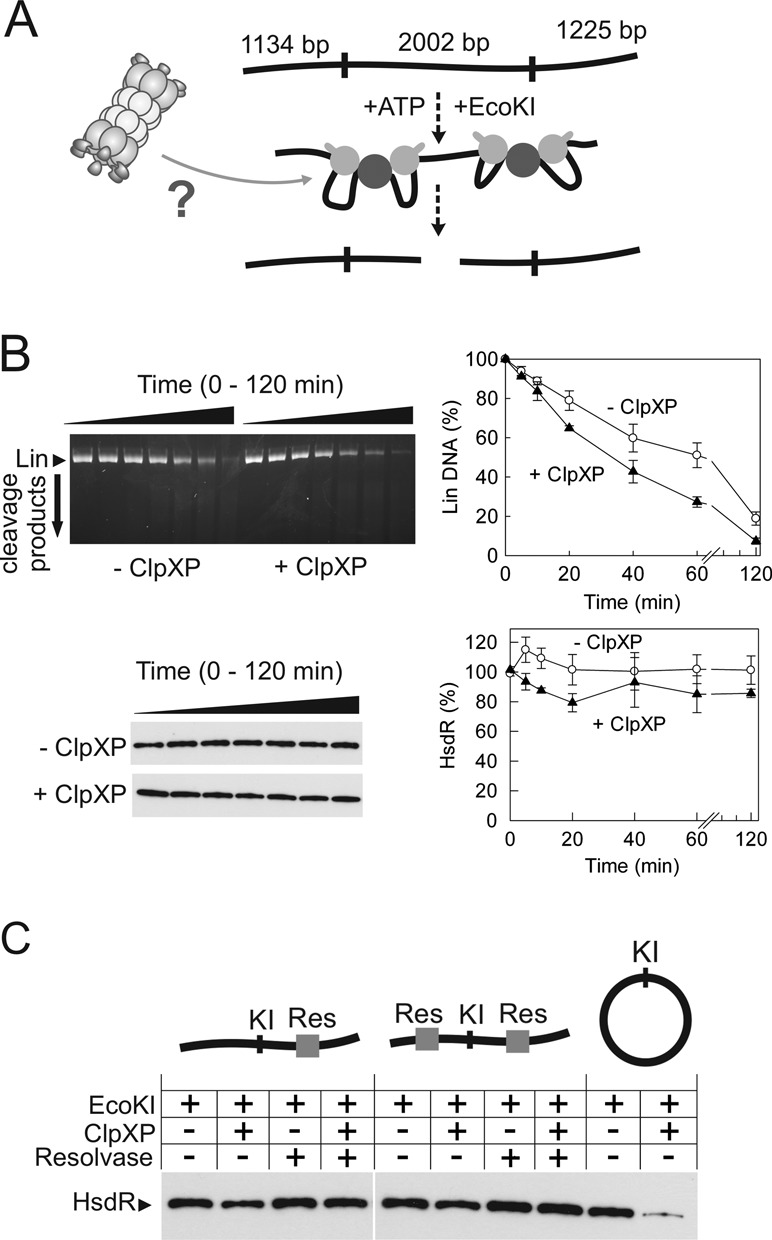
Neither translocation on linear DNA nor collision with static roadblocks stimulates ClpXP degradation of EcoKI HsdR. (**A**) Linear DNA substrate used in the study. Translocation by two EcoKI holoenzymes is required for DNA cleavage. (**B**) Cleavage of the linear DNA by EcoKI was monitored as a function of time (0, 5, 10, 20, 40, 60 and 120 min) in the absence or presence of ClpXP. Samples were separated by either agarose gel electrophoresis (upper gel) or by SDS gel electrophoresis followed by western blotting using an HsdR antibody. Graphs to the right of each gel show quantified data: standard deviation error bars from at least two repeat experiments. (**C**) Proteolysis of HsdR was monitored following 120-min EcoKI reactions in the absence or presence of ClpXP on the DNA shown. For the linear DNA with *res* binding sites, Tn*21* resolvase was also added where indicated. Each *res* site has three binding sites for three resolvase dimers. Samples were separated as in (B).

We have shown previously that termination of Type I RM translocation leads to rapid dissociation of HsdR from linear DNA but only very slow dissociation from circular DNA (53). We suggested that the lifetime of the translocating species is elevated on circular DNA relative to linear DNA. To examine how this difference might affect interactions with ClpXP, we repeated the DNA cleavage and western blot assays using a linear DNA with two EcoKI sites (Figure 5A). A single EcoKI site would not support cleavage of linear DNA, as each HsdR would dissociate from the DNA ends and never communicate in 1-dimension. At least two holoenzymes must be translocating, and collide, to activate the nuclease activity, with cleavage occurring on average midway between the recognition sites (13).

In contrast to results on the circular DNA, ClpXP did not inhibit the cleavage reaction on the linear DNA (in fact, there was a slight increase in cleavage rate) and did not degrade the HsdR within error (Figure 5B). As noted above, the long-lived interaction between HsdR and the cleaved DNA does not result in HsdR degradation, suggesting a different post-cleavage conformational state that is not recognized by ClpXP. The rate of linear DNA cleavage under the PD buffer conditions was actually slower than the rate of circular DNA cleavage. Therefore, the time it takes to cleave the DNA does not appear to be directly relevant to whether or not ClpXP can interact with HsdR. Instead it is more likely that the important factor is the total time in the translocation state (rather than dissociated or cleaved states), which is elevated on circular DNA (53).

### Collision/stalling at protein roadblocks by EcoKI does not induce HsdR degradation by ClpXP

One possible explanation for why the host genomic DNA produces RA and foreign DNA does not is that the density of nucleoprotein complexes on the host DNA may be greater. In the presence of roadblocks, EcoKI may stall numerous times and spend longer in the translocation state before DNA dissociation or cleavage can occur. Whilst our results on circular DNA suggest that protein-dependent stalling is not a prerequisite, conformational changes during stalling with roadblocks may nonetheless increase the chance of ClpXP interaction and increase RA efficiency. To test this, we analysed HsdR degradation on linear DNA in the presence of a stable site-specific roadblock (Tn*21* resolvase bound to its *res* site; Figure 5C). We used two different linear one-site DNA substrates, with either one or two *res* sites present on the translocation path. As expected, neither of the one-site linear DNAs were cleaved by EcoKI (data not shown). The western blot assay shows that HsdR degradation is not activated by collisions with resolvase. One site circular DNA without *res* sites was used as a control, with the expected activation of HsdR degradation. Several other non-specific protein roadblocks were also tested on the one-site linear DNA [the nucleoid-associated proteins StpA and HNS], none of which showed any clear activating effect on HsdR degradation (data not shown).

## DISCUSSION

### ClpXP recognizes long-lived translocation states of HsdR

We demonstrate *in vitro* that ClpXP will proteolyze the HsdR subunit of EcoKI during site-specific DNA translocation, independent of DNA cleavage (Figures 2–4), which is consistent with the proposed model for ClpXP-dependent RA (Figure 1) (42). No other adaptors molecules appear to be necessary for this interaction other than those included in our *in vitro* assays. We were not able to demonstrate complete degradation of HsdR or complete inhibition of DNA cleavage (Figure 2). We suspect that this may reflect the difficulty in finding *in vitro* conditions that are suitable for the robust activity of both enzyme systems. Under our *in vitro* assembly conditions, almost every HsdR subunit should be part of a holoenzyme complex and thus available for interaction with ClpXP (9,53). However, we cannot rule out that partial disassembly in the modified buffer caused release of orphan HsdR subunits which thus became resistant to ClpXP. Proteolysis of HsdR was not observed following DNA cleavage, despite the fact that the HsdRs can remain tightly associated with the cleaved ends and continue to hydrolyze ATP. This may be because there is another change in protein conformation that shields the degron after DNA cleavage. Alternatively, the MTase may act as an adaptor that interacts with ClpX without being a substrate for unfolding. The loss of the MTase following cleavage (53) will therefore prevent ClpX interaction with the orphan HsdR.

In contrast, we did not observe HsdR proteolysis during translocation and/or cleavage of linear DNA substrates. This is not due to a faster linear DNA cleavage rate by EcoKI. This is also unlikely to reflect topological differences between the substrates; we note that foreign circular DNAs, such as M13, are efficiently restricted and do not induce RA to any measurable extent (26). Instead we suggest that the difference between circular and linear DNA *in vitro* reflects a difference in relative ‘translocation lifetime’. We previously showed HsdR motors could ‘turnover’ more rapidly on linear DNA than circular DNA *in vitro*, most likely due to dissociation from the free DNA ends (53). The longer lifetime on circular DNA could give ClpXP a bigger ‘window of opportunity’ to interact compared to linear DNA. Similarly, Makovets *et al.* (33) previously suggested that the delay between recognition of an unmodified site on the genome and DNA damage (see below) would allow recognition by ClpXP. Presumably this ‘window of opportunity’ is far shorter on bacteriophage DNA, most likely due to the higher frequency of unmodified sites per unit length which rapidly leads to DNA cleavage following DNA recognition. We also demonstrated that stalling of the motor at a protein roadblock *in vitro* did not elevate the translocation lifetime sufficiently to activate HsdR proteolysis (Figure 5). Therefore differences in DNA packaging between host and foreign DNA may be less relevant. A definitive answer to why foreign DNA does not activate RA really requires the direct observation of translocation events, and even RA, in living cells.

Some Type I enzymes such as EcoR124I (Type IC) display ClpXP-independent RA (33). In this case, there is no proteolysis and the total levels of HsdR remain constant. Instead the pool of free HsdR available to form the R_2_M_2_S_1_ holoenzyme (necessary for efficient translocation and cleavage) appears to become diluted by the translocation events on the genome. Mutations within EcoR124I HsdR which reduce RA also affect the translocation efficiency by increasing turnover (64). This suggests that translocation lifetime may be a general feature that is used during all types of RA, albeit in different ways (see below).

### Attempts to identify the HsdR degron were unsuccessful

A key question in the ClpXP-dependent RA scheme (Figure 1) is the identity of the degron recognized by ClpX. Our attempts to address this are described in the Supplementary data. Unfortunately, point mutations at putative motifs within the N- and C-termini and random mutations within HsdR did not result in clear phenotypes in either the *in vitro* ClpXP assays or *in vivo* transformation screens. A number of studies have pointed to more complex divalent degrons (45–47), and it is possible that the HsdR interaction requires the sole or di/multivalent use of an internal degron. Proteomics studies that targeted ClpXP substrates using kinetically trapped ClpP complexes from lysates of *E. coli* (36) did not identify HsdR as a substrate. However, if RA was not activated, ClpXP would not necessarily bind to HsdR. Further studies are required to identify the HsdR degron and to show how it is revealed during translocation.

### DNA translocation is potentially toxic but allows an additional level of control

If RA is an effective tool against self-cleavage, why has RA not been observed for other classes of RM enzyme, many of which are related to Type I enzymes? For example, the Type IIB systems such as BcgI share many features (2,65): they also use the same protein complex for both recognition and methylation, making control more difficult; they have target recognition domains similar to HsdS and recognize bipartite DNA sequences; the MTase and nuclease domains are structurally homologous to HsdM and the RecB-like nuclease domain of HsdR, respectively; and the subunits can associate with similar holoenzyme stoichiometry (i.e. two M and R domains per S). However, Type IIB enzymes do not appear to be susceptible to RA. A point mutant (Y439A) in BcgI results in an R+M− mutant that could cleave DNA but could not methylate it (66). Expression of this mutated form of BcgI from a constitutive promoter was lethal to the host, presumably due to the digestion of host DNA. Equivalent mutants of Type I enzymes lead to RA (24,33). The key difference between the Type I and IIB systems is the ATP-dependent DNA translocation of the former catalyzed by helicase domains which the latter do not have. We suggest that a nucleotide triphosphate-dependent motor domain is the key to RA.

Our results with EcoKI show that, even in the absence of DNA cleavage, unregulated DNA translocation (and associated motor stall states; (63)) can be toxic to a cell (Figure 4). The most likely toxic effect of a motor would be due to collision with a replication fork (67), although other processes such as transcription might also be disrupted. Presumably the EcoKI HsdR motors have evolved to be sufficiently stable during translocation that interactions on foreign DNA have a high chance of successfully completing long-range interactions and thus DNA cleavage. The price paid for this is that any translocation events on the host DNA are robust and thus more likely to be disruptive. Nonetheless, to be consistent with the ‘window of opportunity’ model for ClpXP interaction (33), there must be a reasonable delay between recognition of an unmodified host site and the subsequent toxic effects of any translocation.

We speculate that RA may have evolved for Type I enzymes to deal with ‘toxic translocation events’ rather than nuclease-induced dsDNA breaks *per se*. Hence RA is specific to Type I systems that catalyze force-generating translocation. A recurring rhetorical question is, why do Type I enzymes bother with extensive ATP hydrolysis for a thermodynamically favourable reaction such as DNA cleavage? One possible answer is that ATP-dependent translocation allows an additional level of control over the system to prevent autotoxicity (68). In particular, the lifetime of the translocation events may play a key role in both ClpXP-dependent and -independent RA, even if the mechanistic details are quite distinct. This is an evolutionary advantage that the Type I enzymes have over other non-motor-based RM systems. As noted before, the widespread distribution of mechanistically equivalent Type I systems amongst prokaryotes (69) is not because they are more complicated, but perhaps because they are more ‘sophisticated’ (42).

## SUPPLEMENTARY DATA

Supplementary Data are available at NAR Online.

SUPPLEMENTARY DATA

## References

[1] Loenen W.A., Dryden D.T.F., Raleigh E.A., Wilson G.G., Murray N.E. (2014). Highlights of the DNA cutters: a short history of the restriction enzymes. Nucleic Acids Res..

[2] Loenen W.A., Dryden D.T.F., Raleigh E.A., Wilson G.G. (2014). Type I restriction enzymes and their relatives. Nucleic Acids Res..

[3] Pingoud A., Wilson G.G., Wende W. (2014). Type II restriction endonucleases-a historical perspective and more. Nucleic Acids Res..

[4] Rao D.N., Dryden D.T.F., Bheemanaik S. (2014). Type III restriction-modification enzymes: a historical perspective. Nucleic Acids Res..

[5] Hoskisson P.A., Smith M.C. (2007). Hypervariation and phase variation in the bacteriophage ‘resistome’. Curr. Opin. Microbiol..

[6] Makarova K.S., Wolf Y.I., Snir S., Koonin E.V. (2011). Defense islands in bacterial and archaeal genomes and prediction of novel defense systems. J. Bacteriol..

[7] Kennaway C.K., Taylor J.E., Song C.F., Potrzebowski W., Nicholson W., White J.H., Swiderska A., Obarska-Kosinska A., Callow P., Cooper L.P. (2012). Structure and operation of the DNA-translocating type I DNA restriction enzymes. Genes Dev..

[8] Titheradge A.J., King J., Ryu J., Murray N.E. (2001). Families of restriction enzymes: an analysis prompted by molecular and genetic data for type ID restriction and modification systems. Nucleic Acids Res..

[9] Dryden D.T.F., Cooper L.P., Thorpe P.H., Byron O. (1997). The in vitro assembly of the EcoKI type I DNA restriction/modification enzyme and its in vivo implications. Biochemistry.

[10] Janscak P., Dryden D.T.F., Firman K. (1998). Analysis of the subunit assembly of the typeIC restriction-modification enzyme EcoR124I. Nucleic Acids Res..

[11] Weiserova M., Janscak P., Benada O., Hubacek J., Zinkevich V.E., Glover S.W., Firman K. (1993). Cloning, production and characterisation of wild type and mutant forms of the R.EcoK endonucleases. Nucleic Acids Res..

[12] Dryden D.T.F., Cooper L.P., Murray N.E. (1993). Purification and characterization of the methyltransferase from the type 1 restriction and modification system of Escherichia coli K12. J. Biol. Chem..

[13] Studier F.W., Bandyopadhyay P.K. (1988). Model for how type I restriction enzymes select cleavage sites in DNA. Proc. Natl Acad. Sci. U.S.A..

[14] Ellis D.J., Dryden D.T.F., Berge T., Edwardson J.M., Henderson R.M. (1999). Direct observation of DNA translocation and cleavage by the EcoKI endonuclease using atomic force microscopy. Nat. Struct. Biol..

[15] McClelland S.E., Dryden D.T.F., Szczelkun M.D. (2005). Continuous assays for DNA translocation using fluorescent triplex dissociation: application to type I restriction endonucleases. J. Mol. Biol..

[16] Prakash-Cheng A., Ryu J. (1993). Delayed expression of in vivo restriction activity following conjugal transfer of Escherichia coli hsdK (restriction-modification) genes. J. Bacteriol..

[17] Bertani G., Weigle J.J. (1953). Host controlled variation in bacterial viruses. J. Bacteriol..

[18] Thoms B., Wackernagel W. (1982). UV-induced allevation of lambda restriction in Escherichia coli K-12: kinetics of induction and specificity of this SOS function. Mol. Gen. Genet..

[19] Thoms B., Wackernagel W. (1983). Expression of ultraviolet-induced restriction alleviation in Escherichia coli K-12. Detection of a lambda phage fraction with a retarded mode of DNA injection. Biochim. Biophys. Acta.

[20] Thoms B., Wackernagel W. (1984). Genetic control of damage-inducible restriction alleviation in Escherichia coli K12: an SOS function not repressed by lexA. Mol. Gen. Genet..

[21] Efimova E.P., Delver E.P., Belogurov A.A. (1988). 2-Aminopurine and 5-bromouracil induce alleviation of type I restriction in Escherichia coli: mismatches function as inducing signals. Mol. Gen. Genet..

[22] Efimova E.P., Delver E.P., Belogurov A.A. (1988). Alleviation of type I restriction in adenine methylase (dam) mutants of Escherichia coli. Mol. Gen. Genet..

[23] Makovets S., Titheradge A.J., Murray N.E. (1998). ClpX and ClpP are essential for the efficient acquisition of genes specifying type IA and IB restriction systems. Mol. Microbiol..

[24] Makovets S., Doronina V.A., Murray N.E. (1999). Regulation of endonuclease activity by proteolysis prevents breakage of unmodified bacterial chromosomes by type I restriction enzymes. Proc. Natl Acad. Sci. U.S.A..

[25] O'Neill M., Powell L.M., Murray N.E. (2001). Target recognition by EcoKI: the recognition domain is robust and restriction-deficiency commonly results from the proteolytic control of enzyme activity. J. Mol. Biol..

[26] Doronina V.A., Murray N.E. (2001). The proteolytic control of restriction activity in Escherichia coli K-12. Mol. Microbiol..

[27] Blakely G.W., Murray N.E. (2006). Control of the endonuclease activity of type I restriction-modification systems is required to maintain chromosome integrity following homologous recombination. Mol. Microbiol..

[28] Prakash-Cheng A., Chung S.S., Ryu J. (1993). The expression and regulation of hsdK genes after conjugative transfer. Mol. Gen. Genet..

[29] Tao T., Bourne J.C., Blumenthal R.M. (1991). A family of regulatory genes associated with type II restriction-modification systems. J. Bacteriol..

[30] Sorokin V., Severinov K., Gelfand M.S. (2009). Systematic prediction of control proteins and their DNA binding sites. Nucleic Acids Res..

[31] Sorokin V., Severinov K., Gelfand M.S. (2010). Large-scale identification and analysis of C-proteins. Methods Mol. Biol..

[32] Loenen W.A., Daniel A.S., Braymer H.D., Murray N.E. (1987). Organization and sequence of the hsd genes of Escherichia coli K-12. J. Mol. Biol..

[33] Makovets S., Powell L.M., Titheradge A.J., Blakely G.W., Murray N.E. (2004). Is modification sufficient to protect a bacterial chromosome from a resident restriction endonuclease. Mol. Microbiol..

[34] Cromie G.A., Leach D.R. (2001). Recombinational repair of chromosomal DNA double-strand breaks generated by a restriction endonuclease. Mol. Microbiol..

[35] Sauer R.T., Baker T.A. (2011). AAA+ proteases: ATP-fueled machines of protein destruction. Annu. Rev. Biochem..

[36] Flynn J.M., Neher S.B., Kim Y.I., Sauer R.T., Baker T.A. (2003). Proteomic discovery of cellular substrates of the ClpXP protease reveals five classes of ClpX-recognition signals. Mol. Cell.

[37] Neher S.B., Flynn J.M., Sauer R.T., Baker T.A. (2003). Latent ClpX-recognition signals ensure LexA destruction after DNA damage. Genes Dev..

[38] Neher S.B., Villen J., Oakes E.C., Bakalarski C.E., Sauer R.T., Gygi S.P., Baker T.A. (2006). Proteomic profiling of ClpXP substrates after DNA damage reveals extensive instability within SOS regulon. Mol. Cell.

[39] Pruteanu M., Baker T.A. (2009). Proteolysis in the SOS response and metal homeostasis in Escherichia coli. Res. Microbiol..

[40] Pruteanu M., Baker T.A. (2009). Controlled degradation by ClpXP protease tunes the levels of the excision repair protein UvrA to the extent of DNA damage. Mol. Microbiol..

[41] Ambro L., Pevala V., Bauer J., Kutejova E. (2012). The influence of ATP-dependent proteases on a variety of nucleoid-associated processes. J. Struct. Biol..

[42] Murray N.E. (2000). Type I restriction systems: sophisticated molecular machines (a legacy of Bertani and Weigle). Microbiol. Mol. Biol. Rev..

[43] Abdelhakim A.H., Oakes E.C., Sauer R.T., Baker T.A. (2008). Unique contacts direct high-priority recognition of the tetrameric Mu transposase-DNA complex by the AAA+ unfoldase ClpX. Mol. Cell.

[44] Levchenko I., Grant R.A., Wah D.A., Sauer R.T., Baker T.A. (2003). Structure of a delivery protein for an AAA+ protease in complex with a peptide degradation tag. Mol. Cell.

[45] Camberg J.L., Viola M.G., Rea L., Hoskins J.R., Wickner S. (2014). Location of dual sites in E. coli FtsZ important for degradation by ClpXP; one at the C-terminus and one in the disordered linker. PLoS ONE.

[46] Hoskins J.R., Wickner S. (2006). Two peptide sequences can function cooperatively to facilitate binding and unfolding by ClpA and degradation by ClpAP. Proc. Natl Acad. Sci. U.S.A..

[47] Hoskins J.R., Yanagihara K., Mizuuchi K., Wickner S. (2002). ClpAP and ClpXP degrade proteins with tags located in the interior of the primary sequence. Proc. Natl Acad. Sci. U.S.A..

[48] van Aelst K., Toth J., Ramanathan S.P., Schwarz F.W., Seidel R., Szczelkun M.D. (2010). Type III restriction enzymes cleave DNA by long-range interaction between sites in both head-to-head and tail-to-tail inverted repeat. Proc. Natl Acad. Sci. U.S.A..

[49] Stanley L.K., Szczelkun M.D. (2006). Direct and random routing of a molecular motor protein at a DNA junction. Nucleic Acids Res..

[50] Hall S.C., Halford S.E. (1993). Specificity of DNA recognition in the nucleoprotein complex for site-specific recombination by Tn21 resolvase. Nucleic Acids Res..

[51] Bolivar F., Rodriguez R.L., Greene P.J., Betlach M.C., Heyneker H.L., Boyer H.W., Crosa J.H., Falkow S. (1977). Construction and characterization of new cloning vehicles. II. A multipurpose cloning system. Gene.

[52] Vipond I.B., Baldwin G.S., Oram M., Erskine S.G., Wentzell L.M., Szczelkun M.D., Nobbs T.J., Halford S.E. (1995). A general assay for restriction endonucleases and other DNA-modifying enzymes with plasmid substrates. Mol. Biotechnol..

[53] Simons M., Szczelkun M.D. (2011). Recycling of protein subunits during DNA translocation and cleavage by Type I restriction-modification enzymes. Nucleic Acids Res..

[54] Peakman L.J., Szczelkun M.D. (2004). DNA communications by Type III restriction endonucleases-confirmation of 1D translocation over 3D looping. Nucleic Acids Res..

[55] Levchenko I., Yamauchi M., Baker T.A. (1997). ClpX and MuB interact with overlapping regions of Mu transposase: implications for control of the transposition pathway. Genes Dev..

[56] Kim Y.I., Burton R.E., Burton B.M., Sauer R.T., Baker T.A. (2000). Dynamics of substrate denaturation and translocation by the ClpXP degradation machine. Mol. Cell.

[57] Flynn J.M., Levchenko I., Seidel M., Wickner S.H., Sauer R.T., Baker T.A. (2001). Overlapping recognition determinants within the ssrA degradation tag allow modulation of proteolysis. Proc. Natl Acad. Sci. U.S.A..

[58] Cajthamlova K., Šišáková E., Weiser J., Weiserova M. (2007). Phosphorylation of Type IA restriction-modification complex enzyme EcoKI on the HsdR subunit. FEMS Microbiol. Lett..

[59] Davies G.P., Kemp P., Molineux I.J., Murray N.E. (1999). The DNA translocation and ATPase activities of restriction-deficient mutants of Eco KI. J. Mol. Biol..

[60] Davies G.P., Martin I., Sturrock S.S., Cronshaw A., Murray N.E., Dryden D.T.F. (1999). On the structure and operation of type I DNA restriction enzymes. J. Mol. Biol..

[61] Davies G.P., Powell L.M., Webb J.L., Cooper L.P., Murray N.E. (1998). EcoKI with an amino acid substitution in any one of seven DEAD-box motifs has impaired ATPase and endonuclease activities. Nucleic Acids Res..

[62] Webb J.L., King G., Ternent D., Titheradge A.J., Murray N.E. (1996). Restriction by EcoKI is enhanced by co-operative interactions between target sequences and is dependent on DEAD box motifs. EMBO J..

[63] Šišáková E., Weiserova M., Dekker C., Seidel R., Szczelkun M.D. (2008). The interrelationship of helicase and nuclease domains during DNA translocation by the molecular motor EcoR124I. J. Mol. Biol..

[64] Seidel R., Bloom J.G., van Noort J., Dutta C.F., Dekker N.H., Firman K., Szczelkun M.D., Dekker C. (2005). Dynamics of initiation, termination and reinitiation of DNA translocation by the motor protein EcoR124I. EMBO J..

[65] Marshall J.J., Halford S.E. (2010). The type IIB restriction endonucleases. Biochem. Soc. Trans..

[66] Kong H., Smith C.L. (1997). Substrate DNA and cofactor regulate the activities of a multi-functional restriction-modification enzyme, BcgI. Nucleic Acids Res..

[67] McGlynn P., Lloyd R.G. (2002). Recombinational repair and restart of damaged replication forks. Nat. Rev. Mol. Cell Biol..

[68] Bickle T.A. (1993). The ATP-Dependent Restriction Endonucleases in Nucleases.

[69] Roberts R.J., Vincze T., Posfai J., Macelis D. (2010). REBASE-a database for DNA restriction and modification: enzymes, genes and genomes. Nucleic Acids Res..

