# Supplementary material for: ClpXP protease targets long-lived DNA translocation states of a helicase-like motor to cause restriction alleviation
Source: Nucleic Acids Res. 2014 Sep 26;42(19):12082–91. doi: 10.1093/nar/gku851 (PMC4231737; doi:10.1093/nar/gku851)
Supplement: SUPPLEMENTARY DATA [file supp_42_19_12082__index.html]

ClpXP protease targets long-lived DNA translocation states of a helicase-like motor to cause restriction alleviation — ClpXP protease targets long-lived DNA translocation states of a helicase-like motor to cause restriction alleviation — SUPPLEMENTARY DATA 

# ClpXP protease targets long-lived DNA translocation states of a helicase-like motor to cause restriction alleviation

## SUPPLEMENTARY DATA

**Files in this Data Supplement:**

- SUPPLEMENTARY DATA
