# Supplementary material for: ClpXP protease targets long-lived DNA translocation states of a helicase-like motor to cause restriction alleviation
Source: Nucleic Acids Res. 2014 Sep 26;42(19):12082–91. doi: 10.1093/nar/gku851 (PMC4231737; doi:10.1093/nar/gku851)
Supplement: SUPPLEMENTARY DATA [file supp_gku851_nar-02231-h-2014-File007.docx]

**Supplementary Information to:**

**ClpXP protease targets long-lived DNA translocation states of a helicase-like motor to cause restriction alleviation**

Michelle Simons^1^, Fiona M. Diffin^1^ and Mark D. Szczelkun^*^

DNA-Protein Interactions Unit, School of Biochemistry, University of Bristol, Bristol, BS8 1TD, UK

*^1^Joint First Authors*

**SUPPLEMENTARY MATERIALS AND METHODS**

### DNA

Oligodeoxyribonucleotides used in this study are listed in Table S1.

Recombinant clones of HsdR(K4D), HsdR(K4R), HsdR(K11R), HsdR(K4D,K11D), HsdR(V13D) and HsdR(∆N4) were produced by either QuikChange mutagenesis or reverse PCR using pRSF-HsdRΔN20 (53), and the proteins expressed and purified as for the wild type HsdR (main section). N-terminal sequencing confirmed the identity of the proteins.

pGEM::GFP-ssrA (57) was supplied by Mihaela Pruteanu and Tania Baker (MIT). pGEM::GFP-ssrA(LAD) and pGEM::GFP (with a stop codon introduced before the ssrA tag) were generated by QuikChange mutagenesis. To generate pGEM::GFP-HsdR: pGEM-GFP-ssrA was cut with StuI and HindIII, and the DNA purified following agarose gel electrophoresis; and, oligodeoxyribonucleotides coding ten C- terminal amino acids of HsdR were annealed, and ligated to the linear pGEM-GFP. This resulted in the ssrA sequence being replaced by the HsdR sequence.

pVMC3 (11) was supplied by Maria Weiserova. pVMC3(∆HsdR) was produced by introducing a single base deletion into pVMC3 by QuikChange mutagenesis, resulting in a stop codon in the HsdR immediately after Arginine-35. pVMC(HsdR-K4D), pVMC(HsdR-K4R), pVMC(HsdR-K11D), pVMC(HsdR-K4D,K11D), pVMC(HsdR-K11R) and pVMC(HsdR-D298E) were generated by QuikChange mutagenesis of pVMC3. To generate pETcoco-RMS: the complete ΔN20 EcoKI operon from pVMC3 was PCR amplified using primers Oli FD359F and Oli FD359R; the PCR product was cleaved with NsiI and NotI, and ligated to the NsiI/NotI linearised pETcoco-1 (Novagen); QuikChange mutagenesis was used to restore the wild type EcoKI sequence. In pETcoco-RMS and its variants, expression of the MTase is driven by the natural *P*_mod_ promoter whilst expression of HsdR is via the T7 promoter of pETcoco. pETcoco-RMS(HsdR-F7D,F9D), pETcoco-RMS(HsdR-V13D), pETcoco-RMS(HsdR-A1170D), pETcoco-RMS(HsdR-K4D,A1170D) and pETcoco-RMS(HsdR-V13D,A1170D) were generated by QuikChange mutagenesis of pETcoco-RMS.

Random HsdR mutants in pETcoco-RMS were generated by error prone PCR using a GeneMorph II EZClone Domain Mutagenesis kit (Stratagene). Primers Oli FD361F and Oli FD361R were used to generate a ΔN20 HsdR Megaprimer and primers Oli FD361F and Oli HsdR-KI(1R) were used to generate a Megaprimer of the first 500 bp of ΔN20 HsdR. In each case conditions were chosen to favour a low mutation frequency (0 – 4.5 mutations per kbp).

### Purification of GFP-ssrA and variants

Purification of the GFP variants was based on the protocol outlined in Ref. (70). *Escherichia coli* JM109 (DE3) cells were freshly transformed with the expression vector pGEM::GFP-ssrA (or a variant), and subsequently grown in 0.5 L of Luria Broth supplemented with 50 μg/mL ampicillin, at 37 °C to an optical density at 600 nm of ~ 0.5. Protein expression was then induced with 0.5 mM IPTG and the cells harvested after 3 hours growth at 25 °C. The cell paste was stored at – 20 °C until needed. The cell paste was resuspended in 40 mL of 20 mM Tris-Cl, 150 mM NaCl, 5 mM EDTA, pH 8.0, and the cells lysed by sonication for 10 second bursts for two min with intermittent cooling on ice (using a 10 mm tip at 40% amplitude and a VCX 750W Vibra-Cell ultrasonic processor, Sonics & Materials, Inc., CT, USA). Cell debris was removed by centrifugation at *r*_av_ = ~30,000 x *g* for 15 minutes at 4°C.

Dry ammonium sulphate was added to the supernatant to 40% (w/v) saturation and incubated at 4 °C for 1 hour. Precipitated proteins were removed by centrifugation at *r*_av_ = ~4,300 x *g* for 20 minutes at 4°C. Ammonium sulphate was added to the supernatant to a final concentration of 70% (w/v) saturation. The solution was added to 1/4 volume 100% (v/v) ethanol, mixed vigorously at room temperature for 1 minute, and phase separation achieved by centrifugation at *r*_av_ = ~4,000 x *g* for 5 minutes at room temperature. The upper organic phase was retained and a 1/16 volume of ethanol added to the lower phase and phase separation carried out as before. The ethanol extracts were pooled and added to a 1/4 volume of butan-1-ol, mixed vigorously and the phases separated as above. The lower aqueous phase containing GFP was added to an equal volume of chloroform, mixed vigorously and the phases separated. The upper phase was retained and an equal volume of 30% (w/v) ammonium sulphate added to the lower phase, mixed and phase separation carried out as before. The upper phase was retained and pooled with the upper phase from the previous step. This was loaded onto a HiPrep 16/10 Phenyl FF (high sub) column (GEHealthcare) equilibrated with 20 mM Tris-Cl, 1 mM EDTA, pH 8.0, 20% (w/v) ammonium sulphate. The column was washed with 20 mM Tri-Cl, 1 mM EDTA, pH 8.0 followed by ddH_2_O, and then the bound GFP eluted with 70% (v/v) ethanol.

Fractions containing GFP were pooled and buffer exchanged into 20 mM Tris-Cl, 1 mM EDTA, pH 8.0, and concentrated using Amicon Ultra-15 centrifugal filter devices (10,000 MWCO) (Millipore). Aliquots were snap frozen in liquid nitrogen and stored at -80 °C. Each sample was considered “single use” and was not re-frozen upon thawing. Protein concentration was determined using the Bio-Rad Protein Assay according to the manufacturer’s instructions, using BSA as a protein standard (Bio-Rad).

### GFP degradation assays

GFP degradation assays were designed based on previous experiments (57). Assays were performed using a Cary Eclipse Varian Fluorescence Spectrophotometer with excitation at 467 nm and emission 510 nm, and slit widths of 5 mm. Reactions were performed in PD buffer (see main section) supplemented with 4 mM ATP, 25 U/mL creatine phosphokinase, 5 mM phosphocreatine, 0.3 μM ClpX and 0.8 μM ClpP. The reaction mix was incubated at 30 °C for 2 minutes and the reaction started by the addition of GFP to 5 μM; recording commenced after 10 seconds and continued for 30 minutes.

### Peptide Array

Peptide array experiments were adapted from Ref. (36). A cellulose membrane containing peptides corresponding to the N-terminus of HsdR and other control proteins was prepared by Richard Cook at the MIT Biopolymers Facility using an Abimed instrument. Each peptide was covalently attached to the membrane via two C-terminal ß alanines and a PEG linker. The membrane was soaked in 100% (v/v) ethanol for 5 minutes, placed on a rocker at 4 °C then washed three times in 20 mL PBS-T [phosphate buffered saline supplemented with 0.1% (v/v) Tween 20] for 5 minutes. The membrane was then blocked overnight in milk solution [10% (w/v) Semi-Skimmed Milk powder (Somerfield) in PBS]. The membrane was washed twice in 10 mL PBS-T for 5 minutes and twice in 10 mL ClpX buffer (50 mM HEPES pH 7.5, 150 mM KCl, 5 mM MgCl_2_, 100 μM ZnSO_4_, 2 mM DTT). 0.8 μM his-ClpX_6_ and 4 mM ATPγS were incubated in 5 mL ClpX buffer at 30 °C for 2 minutes then added to the membrane with 0.1% (w/v) Milk and incubated for 6 hours. The membrane was then washed three times in 5 mL ClpX buffer supplemented with 0.5 mM ATPγS for 5 minutes. The anti His antibody [6x His Monoclonal (BD Biosciences) at 0.52 mg/mL] was diluted 5000-fold in 5 mL of ClpX buffer supplemented with 1 mM ATPγS, and incubated with the membrane for 30 minutes. The membrane was washed three times in 5 mL ClpX buffer supplemented with 0.5 mM ATPγS for 5 minutes. 5 mL of a 1:5,000 dilution of goat anti-mouse HRP conjugated antibody (Santa Cruz Biotechnology) plus 1 mM ATPγS was incubated with the membrane for 20 minutes. Finally, the membrane was washed three times in 5 mL ClpX buffer supplemented with 0.5 mM ATPγS for 5 minutes.

To generate a chemiluminescent signal, Roche POD Western Blotting substrate was used according to manufacturer’s instructions. Visualization was performed by exposing Hyperfilm MP (Amersham) to the blot, before developing the film with an Agfa Curix 60 film processor. The developed peptide arrays were scanned and analysed using the Analysis Toolbox software of ImageQuant (Amersham Biosciences, ImageQuant TL version 2005).

**Transformation survival screens**

**Screen 1:** Samples of electrocompetent *E. coli* NK311 [*Δ*(*hsdRMS*)] or NK312 cells [*Δ*(*hsdRMS*) *ΔclpX*] (24), were separated into two aliquots. The first aliquot was transformed with 10 ng pBR322 (51) and the second aliquot with 10 ng pVMC3 or pVMC3 mutants. Transformants were selected on Luria Broth agar supplemented with 100 µg/ml ampicillin for pVMC3 based plasmids or with 10 µg/ml tetracycline for pBR322. For the NK312 strain, plates were additionally supplemented with 50 µg/ml kanamycin to maintain the transposon mutation of *clpx*. Serial dilutions were made and duplicate aliquots (100 µl) of diluted and undiluted transformations were spread onto selective solid media and grown at 37 °C for >12 hours. Colonies were counted, and the average number of c.f.u. / 100µl of each transformation calculated.

**Screen 2:** Samples of electrocompetent *E. coli* NK311 or NK312 were separated into two aliquots. The first aliquot was transformed with 10 ng pETcoco-1 and the second aliquot with 10 ng pETcoco-RMS or pETcoco-RMS mutants. Transformants were selected on Luria Broth agar supplemented with 17 µg/ml chloramphenicol and either 0.2% (w/v) D-glucose (plasmid at single copy and HsdR expression repressed) or 0.01% (w/v) L-arabinose and 1mM Isopropyl β-D-1-thiogalactopyranoside (IPTG) (plasmid at medium copy, 20-30 per cell, and HsdR expression by *E. coli* RNA polymerase transcription from the T7 promoter). For the NK312 strain, plates were additionally supplemented with 50 µg /ml kanamycin to maintain the transposon mutation of *clpx*. Serial dilutions were made and duplicate aliquots (100 µl) of diluted and undiluted transformations were spread onto selective solid media and grown at 37 °C for >12 hours. Colonies were counted, and the average number of c.f.u. / 100µl of each transformation calculated.

**Screen 3:** To select for loss of ClpX interaction using random mutations in HsdR, pETcoco-RMS DNA from the PCR random mutagenesis were used to transform electrocompetent *E. coli* NK311. Cells were plated onto Luria Broth agar supplemented with 17 µg/ml chloramphenicol and 0.2% (w/v) D-glucose (plasmid at single copy and HsdR expression repressed) at a density to form single isolated colonies. The colonies were then replica-plated onto Luria Broth agarose supplemented with 17 µg/ml chloramphenicol, 0.01% (w/v) L-arabinose and 1 mM Isopropyl β-D-1-thiogalactopyranoside (plasmid at medium copy, 20-30 per cell, and HsdR expression by *E. coli* RNA polymerase transcription from the T7 promoter). Growth on the replica plate was taken as evidence for RA. Hence, colonies were screened for loss of growth (or poor growth scored as pin-prick colonies) as a readout of loss of ClpX interaction.

**SUPPLEMENTARY RESULTS**

ClpXP degrons are typically located at the extreme N- or C-termini of ClpX substrates (35,36). The terminal peptide sequences of EcoKI HsdR are shown in Figure S1A. Proteomics and biochemical analysis have identified three possible classes of N-terminal motif and two possible classes of C-terminal motif (Figure S1B) (35,36). Common features of all the motifs are positively charged and/or hydrophobic amino acids; an experimental strategy to prevent ClpX binding is to introduce an acidic residue within these regions (see below). To try to identify residues in HsdR to mutate and test in our ClpXP assay, we first used amino acid sequence comparison. In addition, we used peptide array screening and a GFP-degradation assay. The residues mutated in this study are underlined in Figure S1A and were identified as follows.

**Identifying potential candidates for the HsdR degron at the N-terminus of HsdR**

A simple alignment of the EcoKI HsdR N-terminal sequence against the three N-terminal consensus motifs indicated partial sequence conservation with N-motif 2; lysine-4 and valine-13 are spaced similarly to two conserved residues, although the register relative to the amino terminus is different (we also note that EcoKI has two potential registers – see below). N-motif 2 is found in membrane-associated or secreted proteins and is similar to the SecA peptide signal sequence (3,6). Since some of the cellular EcoKI holoenzyme is found associated with the inner membrane, this is a possibility, although HsdR does not have a strong homology motif characteristic of a signal peptide sequence and the interaction may be via DNA (71).

We also considered the possibility that since the Type I sub-families are related at an amino acid sequence level and that subunit complementation can occur within families (8), other Type IA enzymes may also demonstrate ClpXP-dependent RA. Type IA HsdR subunits can be classified as those with >45% sequence identity with EcoKI HsdR and a corresponding >37% sequence identity with between the HsdM subunits (8). Based on these criteria and using BLAST (http://blast.ncbi.nlm.nih.gov/Blast.cgi), we identified 74 putative members of the Type IA family. The HsdR subunits were aligned at the N-terminus, and a representative subset of this alignment is shown in Figure S1C. There was no evidence for family-wide conservation of the N-motif 2 K(X_8_)V residues seen in EcoKI. Instead we identified an alternative triad of conserved residues. The first conserved cluster had quite variable separation from the putative N-terminus (1 – 28 aa), but we decided to test this as part of a possible degron by mutating phenylalanine-7 and phenylalanine-9 (Figure S1A).

To directly test the interactions of ClpX with the HsdR N-terminal residues, we used a peptide array mapping technique to probe isolated peptide sequences (36). In brief, peptides of twelve amino acids were directly synthesised onto a cellulose membrane via a PEG linker. The membrane was then probed with his-tagged ClpX, washed, and any stable peptide-ClpX interactions detected by immunoblotting (Materials and Methods). The technique was validated using Dps and λO from the N-motif 1 family, IscS and OmpA from the N-motif 2 family and DksA from the N-motif 3 family (data not shown) (36). To test EcoKI HsdR, we first did a sequence scanning experiment, in which the EcoKI N-terminus was examined in packets of 12 amino acids, with each peptide shifted in register by one amino acid (Figure S2A). We used the complete Δ19 sequence of EcoKI HsdR. Therefore, the first peptide represents the N-terminus of Δ19 HsdR whilst the second peptide represents the N-terminus of Δ20 HsdR. As a control, we also tested peptides based on the N-terminal sequences of EcoAI and EcoR124I. Although EcoAI shows some requirement for ClpXP during RA (23), it does not appear to share any of the potential motifs identified for EcoKI. EcoR124I does not require ClpXP for RA (33), and was included as a negative control.

There were strong interactions with three EcoKI-based peptides, which cover the 12 amino acids at the N-terminus of Δ19 HsdR, the 12 amino acids at the N-terminus of Δ20 HsdR, and the 12 amino acids at the N-terminus of the Δ21 sequence (Figure S2A). This flexibility in register could be consistent with the production of both Δ19 and Δ20 forms of HsdR in the cell. There were no convincing interactions between the EcoAI- or EcoR124I-based peptides.

The array approach was extended to analyse mutants of the second EcoKI peptide sequence, corresponding to Δ20 HsdR (Figure S2B). The data was quantified and is presented as a heat map where each row represents a series of peptides where one residue (marked with an asterisk) was replaced with each of the twenty amino acids in turn (the wild type residue is marked with a white X). Each column therefore shows the effect of one particular amino acid when substituted at each position; for example, the substitution of acidic residues generally produced a general negative effect at each position. We note that there was variation in the relative response of the wild type sequence, which may reflect variations in the signal across the blot, due in part to variations in the efficiency of *in situ* peptide synthesis. Two residues that were relatively intolerant of change were lysine-4 and lysine-11, which showed decreased intensity across each respective row except where the wild type residue was exchanged conservatively for a basic arginine. Therefore, in addition to lysine-4 identified as a putative N-motif 2 above (Figure S1A), we also made mutations at lysine-11. We decided to further test the importance of the lysine residues by making an additional N-terminal truncation (∆N4, Figure S1A) that removes lysine-4 and shifts the register. The equivalent peptide did not interact in the peptide scanning experiment (Figure S2A). In comparison to the lysine residues, phenylalanine-7 and phenylalanine-9 identified from the Type 1A alignments (Figure S1C), and valine-13 identified as part of a putative N-motif 2 (Figure S1B), appeared to be more tolerant of non-conservative substitutions. Nonetheless, we also made mutants at valine-13 to further test the putative N-motif 2.

**Identifying potential candidates for the HsdR degron at the C-terminus of HsdR**

On the basis of a simple alignment, the C-terminus of EcoKI showed similarity to C-motif 1 (Figure S1A,B), although the consensus is somewhat redundant. This motif includes the SsrA tag that is added to polypeptides that stall during synthesis on the ribosome (72). However, EcoKI lacks elements of the full tag sequence (AANDENYALAA) that also includes recognition elements for the adaptor protein SspB (35), and we did not find any strong sequence conservation at the C-terminus of the Type IA family HsdRs (Figure S1D).

To test the C-terminal HsdR sequence indirectly, we used a GFP degradation assay (57). GFP is normally not degraded by ClpXP even following hours of incubation and this can be monitored by the stability of GFP fluorescence. However, a chimera with a C-terminal SsrA tag is slowly degraded by ClpXP with concomitant loss of florescence signal (Figure S2C, *left panel*). As controls we used two variants of GFP-ssrA; GFP-ssrA(LAD) and GFP. The mutation of the terminal alanine residue to aspartic acid prevents degradation, whilst untagged GFP is not a substrate for ClpXP (Figure S2C, *middle panel*). When we added the final ten amino acids of the EcoKI HsdR sequence (aa 1161-1170) to the C-terminus of GFP, no change in the fluorescence signal was observed. This suggested that the C-terminus of HsdR alone was not a robust ClpX degron. Nonetheless, some ClpXP targets rely on sequence at both ends of the protein (73). We therefore made C-terminal mutations at alanine-1170, either singly or in combination with residues identified at the N-terminus (see below).

**Testing mutations in the putative HsdR degron(s) *in vitro***

We first used the *in vitro* DNA cleavage/Western blot assays described in the main section to test a representative selection of the HsdR mutants. EcoKI holoenzyme, ClpXP and pLKS5 were incubated for one hour and the extent of DNA cleavage (as percentage of scDNA remaining) and HsdR proteolysis, quantified (main section Materials and Methods) (Figure S3A). For a reaction to be equivalent to wild type, we expected ~80% scDNA cleavage in the absence of ClpXP and ~60% in its presence, with a corresponding proteolysis of HsdR of greater than ~60% (*viz*. Figure 4A).

Although replacement of lysine-4 with aspartic acid had a strong phenotype in the peptide array (Figure S2B), the DNA cleavage and HsdR levels with both K4D and ΔN4 appeared similar to wild type. The double mutant K4D,K11D and conservative substitutions at each residue with arginine also gave results consistent with a wild type interaction. In contrast, aspartic acid substitutions at valine-13 and at alanine-1170 showed phenotypes consistent with a ClpX interaction – ClpXP did not inhibit DNA cleavage and HsdR was not degraded. This suggested that interactions with valine-13 and/or alanine-1170 may be occurring. An alternative explanation for these results is that the V13D and A1170D mutations reduce the DNA-interaction lifetime of the HsdR subunits following translocation termination, so that these reactions are more equivalent to those on linear DNA where ClpXP has no effect (Figure 5).

**Testing mutations in the putative HsdR degron(s) *in vivo***

Since the HsdR-ClpX interaction is likely to be more robust *in vivo*, we decided to verify our testing of the HsdR mutants using transformation survival screens (Figure S3B,C) (23). The basic principal of these assays is that naïve strains (i.e. hsdRMS-) which are also either Clp+ (NK311) or Clp- (NK312) are transformed with plasmids carrying the complete EcoKI operon, and single colonies are counted on solid media selective for antibiotic resistance carried by the plasmid. Using plasmids expressing wild type EcoKI, there would be significantly fewer successful transformants using the Clp- strain compared to the Clp+ strain because, in the absence of RA, the holoenzyme will form and cleave the DNA of the naïve host. Mutants that interfere with the HsdR-ClpX interaction will also interfere with RA and there will be fewer transformants observed on both the Clp+ and Clp- strains.

We used two versions of the assay (Supplementary Materials and Methods). In the first version (Screen 1, Figure S3B), cells were transformed with either pVMC3 (which carries the EcoKI operon controlled by *P*_res_ and *P*_mod_) or pBR322 (the parental plasmid of pVMC3). The efficiency of transformation was scored as the ratio of the c.f.u. for pVMC3 relative to the c.f.u. for pBR322. We found that some mutations were difficult to establish in the moderately high copy number pVMC3, so we switched to a second version of the assay where holoenzyme expression was tunable (Screen 2, Figure S3C). The EcoKI operon was cloned into the plasmid pET-coco-1 to produce pETcoco-RMS, where *hsdr* was under control of *P*_t7lac_. Low level expression from this promoter is possible by *E. coli* RNA polymerase (74). We controlled expression in two ways: Firstly, Lac repressor binding at *P*_t7lac_ prevented HsdR expression unless the inducer (IPTG) were added; Secondly, the pET-coco plasmid has two replication origins (oriS and oriV) and was maintained at single-copy number by propagation in Luria Broth plus glucose. Copy number amplification (to ~20-30 per cell) was achieved by inducing the TrfA replicator under the control of the araC-*P*_BAD_ promoter system using arabinose. Each strain was transformed with pETcoco-RMS or pET-coco-1. Aliquots were spread onto solid media with either glucose (single copy number, HsdR “off”) or arabinose plus IPTG (medium copy number, HsdR “on”), and grown at 30 °C for >12 hours. Efficiency was scored as the c.f.u. ratio for pETcoco-RMS against pETcoco-1.

In both screens, ClpXP significantly enhanced the transformation efficiency when using wild type EcoKI (Figure S3B,C). In screen 1 (panel B), amino acid changes at lysine-4 and lysine-11 had little effect, with ClpXP enhancements similar to wild type. These results are consistent with the *in vitro* assay (Figure S3A) and suggest that the results observed with the peptide array (Figure S2B) may be specific to the isolated peptides and not the full length protein. As a control we also used tested an HsdR endonuclease mutation (D298E, Nuc-). We expected this to show a smaller ClpXP enhancement than wild type as Nuc- would be less toxic to the cells. Instead the ClpXP enhancement was at least the same, if not greater, than with wild type HsdR, suggesting that DNA translocation by Type I enzymes is toxic even in the absence of DNA cleavage (see main text).

To test valine-13 (putative N-motif 2) and alanine-1170 (putative C-motif 1), we used the alternative screen 2 (Figure S3C). The A1170D mutant had a ClpXP enhancement value similar to wild type, suggesting that any bypass of the ClpXP interaction in the *in vitro* assay (Figure S3A) is not significant *in vivo*. In contrast, the V13D mutant had a markedly reduced ClpXP enhancement value (by >5000-fold). However, this was not due to a decrease in transformation efficiency on the Clp+ strain but due rather to an increase in transformation efficiency on the Clp- strain (although the colonies had a distinct, small phenotype). In other words, the V13D mutation produces an EcoKI holoenzyme that is less toxic than wild type during transformation and therefore does not appear to require ClpXP for RA. A similar result was also observed when the A1170D mutation was combined with either the V13D or K4D mutation, and also with the F7D,F9D double mutant.

**Testing random mutations in HsdR *in vivo***

Random mutations were introduced either throughout *hsdr* or restricted to the first 500 bp of *hsdr* in pETcoco-RMS by error-prone PCR using a GeneMorph II EZClone Domain Mutagenesis kit (Stratagene). Sequencing analysis showed that, on average, 2.3 mutations/kb had been introduced. The resultant PCR products were transformed into ClpX+ cells (NK311) and replicated-plated colonies screened for loss of growth (score as no visible colony) or poor growth (scored as a small colony) on solid media that causes HsdR overexpression. Poor growth upon over-expression of HsdR in a ClpXP background can be taken as an indicator of loss of ClpX interaction. In all, >11,000 colonies were screened but we were unable to identify any candidates for further analysis.

**SUPPLEMENTARY CONCLUSIONS**

Despite similarities in the sequence of HsdR EcoKI with previously identified ClpX motifs at both the N- and C-termini (Figure S1), we were not able to characterise any mutation within those regions which led to a clear loss of EcoKI-ClpX interaction. Lysine-4, lysine-11, phenylalanine-7 and phenylalanine 9 all gave wild type results in the *in vitro* and/or *in vivo* tests. The noticeable effect of non-conservative substitutions at lysine-4 and lysine-11 in the peptide array may reflect strong ClpX interactions with basic residues, rather than any HsdR-specific motif. Valine-13 and alanine-1170 showed clearer candidate phenotypes based on the *in vitro* DNA and protein cleavage assays. However, alanine-1170 gave a wild type phenotype in the transformation screen and the tagging of GFP with the C-terminus of HsdR also gave a negative result. Valine-13 gave a reduced ClpXP enhancement in the transformation screen, but this was due to a reduced toxicity in the ClpX- strains, suggesting that RA was not as important for this mutant. This *in vivo* effect was also observed with other mutations or combinations of mutations which gave wild type results in the other assays. This includes phenylalanine-7 and -9, further suggesting that the Type IA family-specific N-terminal motifs (Figure S1C) are not necessary for ClpXP interaction.

Since the HsdR degron may be bivalent or even multivalent, or may rely on internal sequences, we also attempted a random mutagenic screen using transformation survival as readout of loss of ClpX interaction. This was unsuccessful. A more systematic analysis of HsdR mutants will be required in the future, using both *in vitro* and *in vivo* screens, if we are to identify the degron.

**SUPPLEMENTARY REFERENCES**

70. Yakhnin, A.V., Vinokurov, L.M., Surin, A.K. and Alakhov, Y.B. (1998) Green fluorescent protein purification by organic extraction. *protein Expression and Purification*, **14**, 382 - 386.

71. Holubova, I., Vejsadova, S., Firman, K. and Weiserova, M. (2004) Cellular localization of Type I restriction-modification enzymes is family dependent. *Biochem. Biophys. Res. Commun.*, **319**, 375-380.

72. Moore, S.D. and Sauer, R.T. (2007) The tmRNA system for translational surveillance and ribosome rescue. *Annu. Rev. Biochem.*, **76**, 101-124.

73. Gonciarz-Swiatek, M., Wawrzynow, A., Um, S.J., Learn, B.A., McMacken, R., Kelley, W.L., Georgopoulos, C., Sliekers, O. and Zylicz, M. (1999) Recognition, targeting, and hydrolysis of the lambda O replication protein by the ClpP/ClpX protease. *J. Biol. Chem.*, **274**, 13999-14005.

74. Manelyte, L., Guy, C.P., Smith, R.M., Dillingham, M.S., McGlynn, P. and Savery, N.J. (2009) The unstructured C-terminal extension of UvrD interacts with UvrB, but is dispensable for nucleotide excision repair. *DNA Repair (Amst)*, **8**, 1300-1310.

### Table S1. Oligodeoxribonucleotides

| Olimsi031 | GAAGGAGATATACATATGAATGATTCCAATTTTGAATTCCTGAAGGGCG | QuickChange mutagenesis  K4D  pRSF-HsdRΔN20 |
| --- | --- | --- |
| Olimsi032 | CGCCCTTCAGGAATTCAAAATTGGAATCATTCATATGTATATCTCCTTC |  |
| Oli#ms294F | GAAGGAGATATACATATGAATAGATCCAATTTTGAATTCCTGAAGGGCG | QuikChange mutagenesis  K4R  pRSF-HsdRΔN20 |
| Oli#ms294R | CGCCCTTCAGGAATTCAAAATTGGATCTATTCATATGTATATCTCCTTC |  |
| Olimsi028 | CATATGTATATCTCCTTCTTATACTTAAC | Deletion of HsdR N-terminus  ∆N4  pSF-HsdRΔN20 |
| Oli #ms298F | TCCAATTTTGAATTCCTGAAG |  |
| Oli FD358F | GAATTCCTGGACGGCGTCAACGACTTCAC | QuikChange mutagenesis  K11D  pRSF-HsdRΔN20(K4D) and pVMC3/pVMC3(K4D) |
| Oli FD358R | GTGAAGTCGTTGACGCCGTCCAGGAATTC |  |
| Oli#ms296F | AAATCCAATTTTGAATTCCTGAGAGGCGTCAACGACTTCACTTATGC | QuikChange mutagenesis  K11R  pRSF-HsdRΔN20 and pVMC3 |
| Oli#ms296R | GCATAAGTGAAGTCGTTGACGCCTCTCAGGAATTCAAAATTGGATTT |  |
| Oli FD363FQC | GAATTCCTGAAGGGCGACAACGACTTCACTTATG | QuikChange mutagenesis  V13D  pRSF-HsdRΔN20 and pETcoco-RMS |
| Oli FD363RQC | CATAAGTGAAGTCGTTGTCGCCCTTCAGGAATTC |  |
| Oli FD366FQC | GACGAGCTGGACTGACTCGAGTCTGGTAAAG | QuikChange mutagenesis  A1170D  pRSF-HsdRΔN20 |
| Oli FD366RQC | CTTTACCAGACTCGAGTCAGTCCAGCTCGTC |  |
| Oli#ms299F | GGGTAATCAGGGCTTTGCGGAATATGTGCTGTTTGTCG | QuikChange mutagenesis  D298E  pRSF-HsdRΔN20 and pVMC3 |
| Oli#ms299R | CGACAAACAGCACATATTCCGCAAAGCCCTGATTACCC |  |
| Oli#ms300F | CGACCGGTACCGGTAGAACCCGTACGGCAATCGC | QuikChange mutagenesis  K477R  pRSF-HsdRΔN20 |
| Oli#ms300R | GCGATTGCCGTACGGGTTCTACCGGTACCGGTCG |  |
| Olimsi010 | CGAAAACTACGCTTTAGCAGATTAATAAGCTTAATTAGCTGAGC | QuickChange mutagenesis  GFP-ssrA (LAD) |
| Olimsi011 | GCTCAGCTAATTAAGCTTATTAATCTGCTAAAGCGTAGTTTTCG |  |
| Olimsi014 | CGTCGTTTGCTGCAGGTCATTTGTATAGTTCATCCATGCC | QuickChange mutagenesis  GFP minus ssrA |
| Olimsi015 | GGCATGGATGAACTATACAAATGACCTGCAGCAAACGACG |  |
| Olimsi016 | CCCTTCAGCGATTATATCTGGGACGAGCTGGCCTGATA | GFP–HsdR cloning |
| Olimsi017 | AGCTTATCAGGCCAGCTCGTCCCAGATATAATCGCTGAAGGG |  |
| Oli#ms301F | GGATTAATAACAATGATGAATGATTCCAATTTTGAATTCCTGAAGGGCG | QuikChange mutagenesis  K4D  pVMC3 |
| Oli new301R | CTTCAGGAATTCAAAATTGGAATCATTCATCATTGTTATTAGTCC |  |
| Oli#ms302F | GGATTAATAACAATGATGAATAGATCCAATTTTGAATTCCTGAAGGGCG | QuikChange mutagenesis  K4R  pVMC3 |
| Oli#ms302R | CGCCCTTCAGGAATTCAAAATTGGATCTATTCATCATTGTTATTAGTCC |  |
| Oli FD359F | GCGTACGGATGCATAAATCCAATTTTGAATTCC | Amplification of EcoKI operon NsiI and NotI ends for cloning into pETcoco-1 |
| Oli FD359R | GCGTAAGTGCGGCCGCTCAGGATTTTTTACGTGAGGCTT |  |
| Oli FD360F | GGAGATATAAGCATGAATAAATCCAATTTTG | QuikChange mutagenesis  Remove NsiI site and restore *hsdr*  pETcoco-RMS |
| Oli FD360R | CAAAATTGGATTTATTCATGCTTATATCTCC |  |
| Oli FD361F | GTTTAACTTTAAGAAGGAGATATAAGCATG | Primer pair to make 'Mega Primer' for Mutazyme II reaction |
| Oli FD361R | CAGCCTGAAGGATGAAGTGTATACGTGTCA |  |
| Oli FD365FQC | GACGAGCTGGACTGACACGTATACACTTC | QuikChange mutagenesis  A1170D  pETcoco-RMS |
| Oli FD365RQC | GAAGTGTATACGTGTCAGTCCAGCTCGTC |  |
| Oli FD364FQC | GGAGATATAAGCATGAATGACTCCAATTTTG | QuikChange mutagenesis  K4D  pETcoco-RMS |
| Oli FD364RQC | CAAAATTGGAGTCATTCATGCTTATATCTCC |  |
| Oli HsdR-KI(1R) | CTCGCACCTGCTGTTCAAG | Used with Oli FD361F to make 'Mega Primer' |
| Oli FD368FQC | GAATAAATCCAATGATGAAGACCTGAAGGGC | QuikChange mutagenesis  F7D, F9D  pETcoco-RMS |
| Oli FD368RQC | GCCCTTCAGGTCTTCATCATTGGATTTATTC |  |


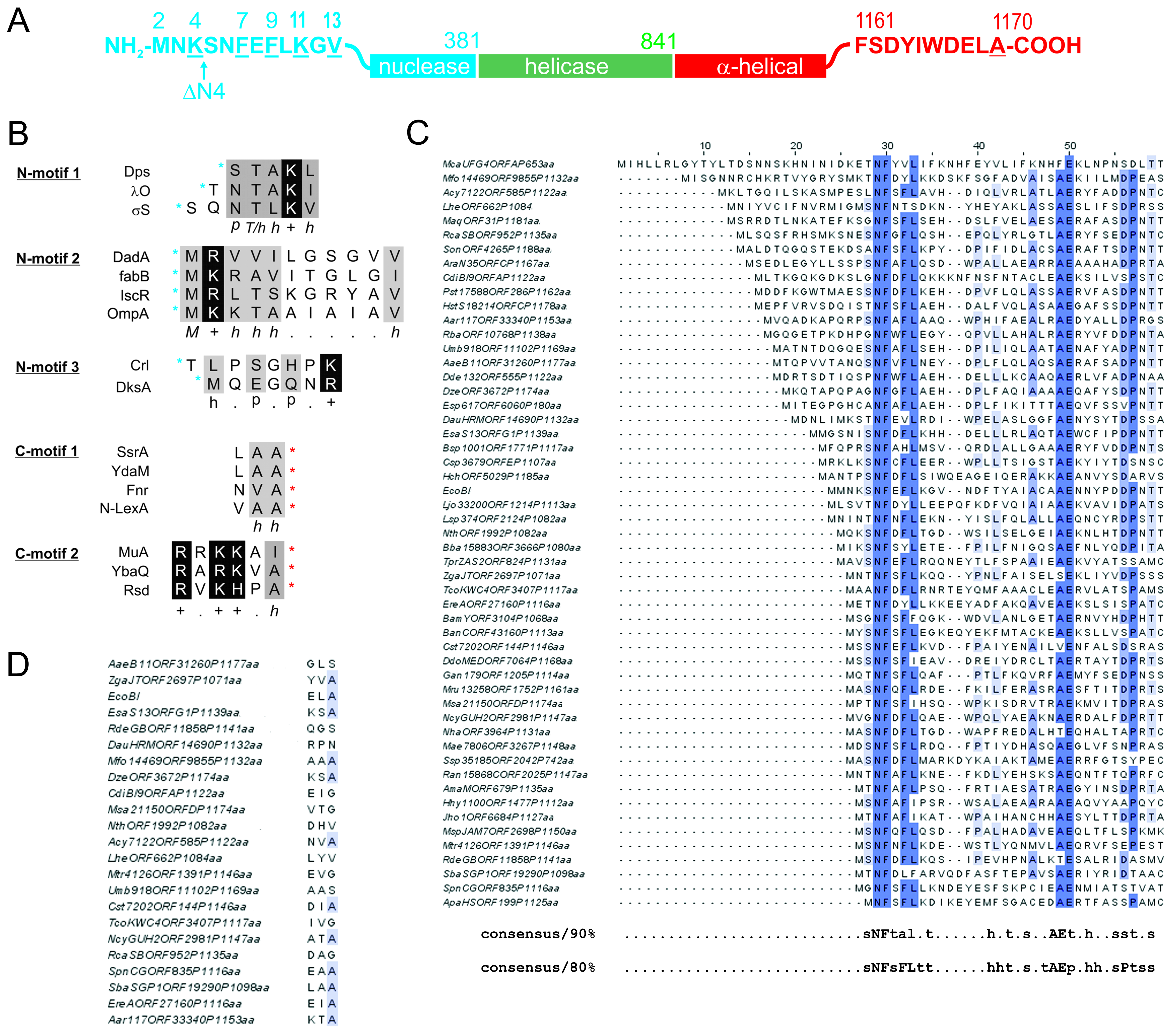


**Figure S1.** Possible degrons in EcoKI HsdR**.** (**A**) Cartoon of EcoKI HsdR showing domains and N- and C-terminal sequences with mutations underlined, and the truncation indicated with an arrow. Domain interfaces are approximate. (**B**) Degron sequences identified from proteomic screening (35,36). Asterisks show the terminal amino (blue) or carboxyl (red) groups. (**C**) Alignment of representative HsdR N-terminal sequences from the Type IA family. EcoKI is not shown but the EcoBI sequence is identical in this region. (C) Alignment of representative HsdR C-terminal sequences from the Type IA family.


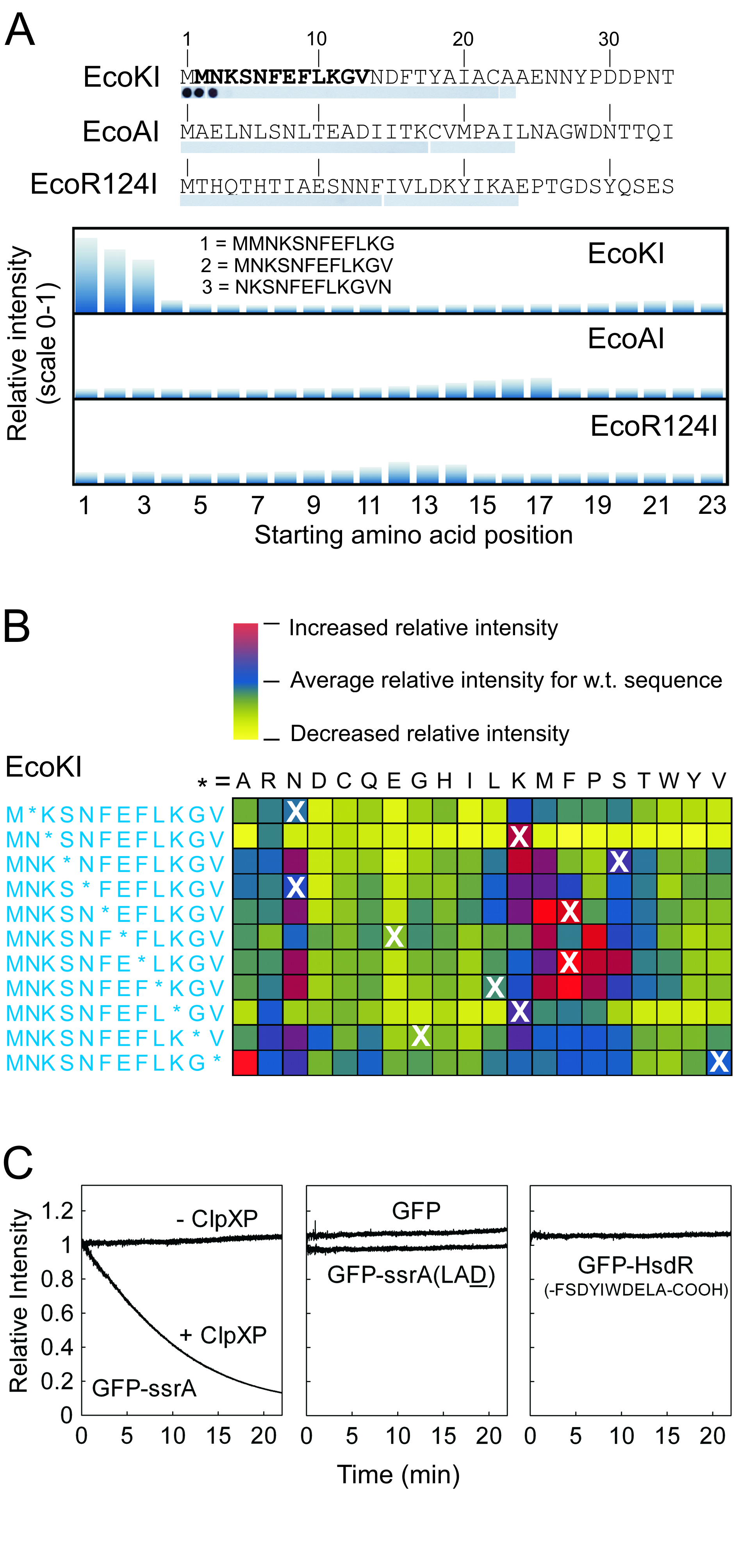


**Figure S2.** *In vitro* assays of HsdR sequences. (**A**) Peptide array for sequences (**Figure S1.** 12 amino acids) derived from the N-termini of EcoKI HsdR, EcoAI HsdR and EcoR124I HsdR. Blots are aligned with the N-terminal amino acid of each 12-mer. For example, the sequences of the first three EcoKI peptides which give signals are given in the graph. The graph shows quantified data from the blots. (**B**) Quantified data from the peptide array for an amino acid scanning experiment. The 12-mer peptide sequence from the N-terminus of EcoKI HsdR was screened for ClpX binding activity with positions 2-12 replaced in turn by each of the 20 possible amino acids. The heat map has a linear (log_2_) false colouring. The wild type sequence is repeated at each location marked by a white X. (**C**) GFP degradation assay. GFP variants were mixed with ClpXP and the fluorescence intensity (shown as a value relative to the starting fluorescence) monitored for 30 minutes.


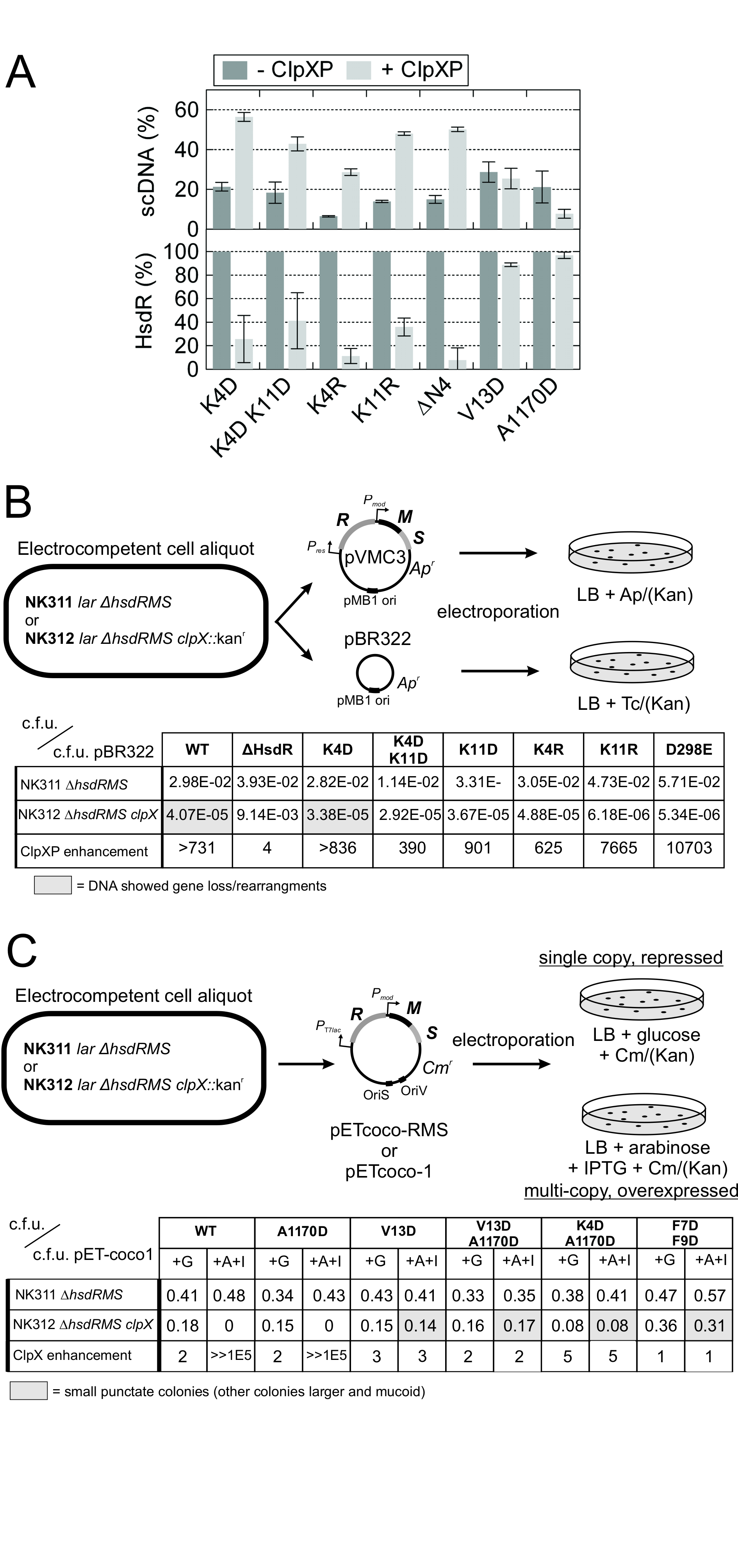


**Figure S3.** *In vitro* and *in vivo* assays for ClpX interaction with wild type and mutant EcoKI. (**A**) The extent of DNA cleavage (measure as supercoiled DNA remaining) and of HsdR proteolysis was measured for 60 minutes for the EcoKI holoenzymes assembled with the HsdRs indicated. Assays are described in the main text. Error bars are standard deviations from at least two repeat experiments. (**B**) Transformation survival Screen 1. (**C**) Transformation survival Screen 1. See Supplementary text for further details.
